# Generation of multicellular tumor spheroids with micro-well array for anticancer drug combination screening based on a valuable biomarker of hepatocellular carcinoma

**DOI:** 10.3389/fbioe.2022.1087656

**Published:** 2022-12-01

**Authors:** Qi Wang, Juan Liu, Wenzhen Yin, Dawei Sun, Zhongsong Man, Shangwei Jiang, Xiufeng Ran, Yuxin Su, Yunfang Wang, Jiahong Dong

**Affiliations:** ^1^ Department of Hepatobiliary and Pancreatic Surgery, The First Hospital of Jilin University, Jilin University, Changchun, China; ^2^ Hepato-Pancreato-Biliary Center, Beijing Tsinghua Changgung Hospital, Tsinghua University, Beijing, China; ^3^ Research Unit of Precision Hepatobiliary Surgery Paradigm, Chinese Academy of Medical Sciences, Beijing, China; ^4^ Clinical Translational Science Center, Beijing Tsinghua Changgung Hospital, Tsinghua University, Beijing, China; ^5^ Department of General Surgery, Xuzhou Central Hospital, Xuzhou, Jiangsu, China

**Keywords:** hepatocellular carcinoma, FGFR4 specific inhibitors, drug combination, 3D cell culture, parthenolide

## Abstract

Hepatocellular carcinoma (HCC) is a highly malignant tumor with a poor prognosis. More than 30% of patients with diagnosed HCC have abnormally high expression of fibroblast growth factor receptor 4 (FGFR4). Currently, clinical trials for a variety of FGFR4-specific inhibitors have started. However, the effect of these inhibitors is not ideal, and it is necessary to find a drug combination to synergistically exert anti-tumor effects. We found strong correlations between FGFR4 and HCC clinicopathological characteristics in the present study. After grouping patients according to FGFR4 expression, the key gene signatures were inputted the drug-gene related databases, which predicted several potential drug candidates. More importantly, to achieve the reliable and high throughput drug cytotoxicity assessment, we developed an efficient and reproducible agarose hydrogel microwells to generate uniform-sized multicellular tumor spheroids, which provide better mimicry of conventional solid tumors that can precisely represent anticancer drug candidates’ effects. Using high content screening, we quickly evaluated the enhanced anti-tumor effects of these combinations. Finally, we demonstrated that Parthenolide is a potential drug that can significantly enhance the clinical efficacy of FGFR4 receptor inhibitors. In general, we offered a new therapeutic way for FGFR4 positive HCC patients.

## Introduction

Statistics show that HCC accounts for 80%–90% of all primary liver cancers (Yang et al., 2019; Sung et al., 2021), with insidious onset and high degree of malignancy. At diagnosis, HCC is usually in an advanced stage of disease, with a 5-year overall survival rate of less than 20% (Yang et al., 2019). Despite continuous improvements in surgical techniques and local treatments, it is estimated that approximately 60% of HCC patients ultimately receive systemic therapy (Llovet et al., 2021; Llovet et al., 2022).

The fibroblast growth factor (FGF) family consists of 18 ligands and four homologous factors. FGF binds to the corresponding FGF receptor (FGFR1-4) and participates in many physiological processes including embryogenesis, angiogenesis, and material metabolism (Turner and Grose 2010; Xie et al., 2020). As one of the major FGF receptors, FGFR4 binds to the fibroblast growth factor 19 (FGF19) ligands and functions with the help of the FGFR4 *β*-klotho (KLB) synergistic co-receptor. With the increased focus on the significance of FGF19-FGFR4-KLB in HCC, this axis will undoubtedly become a research hotspot (Subbiah and Pal 2019). It is worth noting that the abnormal expression of FGFR4 is closely related to the occurrence and development of HCC (Raja et al., 2019). There are numerous FGFR4 inhibitors on the market, some of which have entered phase I/II clinical trials (Supplementary Table S1). At the moment, the two specific inhibitors making the most progress research are FGF401 (Roblitinib) and BLU-554 (Fisogatinib), both of which have partially entered phase II clinical trials. The phase I/II trial of FGF401 revealed that the effective rate was only 8%, after analyzing 53 FGFR4 positive HCC patients (Stephen L. et al., 2017). The phase I trial of BLU-554 on 66 evaluable FGFR4/FGF19 positive patients yielded a response rate of only 17% (Kim R. D. et al., 2019). In patients with advanced HCC, BLU-554 demonstrated clinical activity comparable to FGF401 (Weiss et al., 2019). In general, a single FGFR4-specific inhibitor has a certain effect on FGFR4 positive HCC, particularly advanced HCC, but it still falls short of ideal expectations.

The successful development of new anti-tumor drugs depends not only on our understanding of their underlying molecular and cellular mechanism, but also on the reliable tumor model for drug evaluation. To achieve a higher success rate in clinical trials, there is an urgent need for the development of drug screening technologies that can predict toxicity and efficacy with greater precision and better depict the tumor microenvironment. Three-dimensional (3D) cell culture systems are one potential option for drug screening applications. Tumor cells in 2D cultures can be stretched, resulting in unwanted cytoskeletal rearrangements and false polarity. In 3D cultures, the cell environment, including cell-cell and cell-matrix interactions, may be replicated with greater fidelity. In our previous work, we have developed a simple but robust human-specific enhanced hepatic spheroid platform based on native liver extracellular matrix (ECM) scaffold with multiparametric readouts to analysis the hepatotoxicity and possible mechanisms induced by drugs (Liu J. et al., 2018; Li R. et al., 2022; Liu J. et al., 2022). Further, based on the invention of microwell array cell spheroid culture plates, we have achieved the high-throughput culturing 3D cell spheroids. With the help of high content imaging, the effect of drug candidates could be quickly assessed.

Hence, in the present work, we concentrated our efforts on identifying the one in the FGFR4-FGF19-KLB axis that is most relevant to the onset and progression of HCC. Following that, we grouped the patients, investigated the variations in gene pathway levels between patients with high FGFR4 expression and those with low expression in HCC, and concluded that FGFR4 may be employed as a major marker target for predicting and treating HCC. Then, in order to address the issue that the effect of FGFR4 inhibitors’ impact is currently difficult to achieve when taken alone, we did a thorough investigation and looked through public databases for suitable combination therapies. The combination of FGFR4 specific inhibitors combined with Parthenolide was found to be the most effective strategy for the treatment of FGFR4 positive HCC patients, which was supported by the effectively 3D cell spheroid experiment evaluation and mechanism exploration, after we obtained the effect of FGFR4 specific inhibitors FGF401 and BLU-554 combined with seven candidate drugs. Our research offers a fresh way for HCC patients with FGFR4 positivity to undergo surgery and increase their survival time.

## Materials and methods

### Databases and analysis methods

The mRNA expression levels of FGFR19, FGFR4 and KLB were validated at GEPIA (Gene Expression Profiling Interactive Analysis) (Tang et al., 2017), which is a newly developed interactive web server for analyzing the RNA sequencing expression data of 9,736 tumors and 8,587 normal samples from the Cancer Genome Atlas (TCGA) and the Genotype-Tissue Expression (GTEx) projects, using a standard processing pipeline (http://gepia.cancer-pku.cn/). The immunohistochemical (IHC) images of FGFR19, FGFR4 and KLB were collected from Human Protein Atlas (https://www.proteinatlas.org/). The mRNA data were juxtaposed with relevant clinicopathological data from the Cancer Genome Atlas database (TCGA, https://portal.gdc.cancer.gov/, updated Mar. 2021). The Limma package in R software (3.6.1. https://www.r-project.org/) based on the negative binomial distribution was used to refine the mRNA data and to identify differentially expressed genes. Differentially expressed genes with a count value of 0 genes were excluded while those with a |log2 fold change (FC)|>1, and *p*-value < 0.05, were considered up-regulated or down-regulated genes. Biological functions of differentially expressed genes were determined by Gene Ontology (GO) enrichment and Kyoto Encyclopedia of Genes and Genomes (KEGG) pathway analysis based on the clusterProfiler, and org.Hs.eg.db package. The GO analysis terms included a cellular component (CC), molecular function (MF), and biological process (BP). Protein-protein Interaction (PPI) network for differentially expressed genes was predicted using the search tool for the retrieval of interacting genes (STRING; http://string-db.org) (Szklarczyk D. et al., 2021) from the online database. Cytoscape bioinformatics software was used to visualize molecular interaction networks. The molecular complex detection (MCODE) method was used to detect molecular complexes in the PPI, and to identify densely connected regions. The prediction of potential drugs were utilized by the cMap (https://clue.io/) (Corsello SM. et al., 2017), which is a database that collects microarray-based gene-expression profiles from cultured human cancer cell lines treated with various experimentally and clinically used small molecules, and provides a pattern-matching Web-based software to mine these data. The more information of drug related targets and pathways were summarized from TTD, (http://db.idrblab.net/ttd/) (Zhou Y. et al., 2022) and Drugbank database (https://go.drugbank.com/) (Wishart DS. et al., 2018).

### Collection of HCC tissues

HCC tissues are obtained from clinical operations, and fresh tissues are fixed and embedded as wax blocks or frozen in liquid nitrogen for the next investigation. All tissues were pathologically confirmed to be HCC, and all patients completed informed consent forms. This study was approved by the ethics committee of Beijing Tsinghua Changgung Hospital.

### Chemicals and reagents

Drug FGF401 was purchased from CASYMCHEM; BLU-554, Geldanamycin, Imatinib, LY-294002, Parthenolide, Tanespimecin, Trichostatin A and Vorinostat were purchased from MedChemExpress. Drug details have shown in Supplementary Table S2. Antibodies FGFR4, p-ERK 1/2 and ERK 1/2 were purchased from Abclonal; Antibodies FGFR4, p-AKT (T308) and AKT were purchased from Cell Signaling Technology; Antibodies FGF19 and KLB were purchased from Abcam. HRP labeled goat anti rabbit IgG (H + L) and HRP labeled goat anti mouse IgG (H + L) were purchased from Beyotime. Antibodies details have shown in Supplementary Table S3.

### Cell culture and cell spheroids formation

HCC cell lines Huh-7, Hep-3B, and HepG2 were cultured in Dulbecco’s modified Eagle’s medium (DMEM) complete medium. Growth media were supplemented with 10% (vol/vol) fetal bovine serum (Gibco) and penicillin-streptomycin (100 U/ml) in a 5% CO_2_-humidified chamber at 37°C. The cells were passaged every 2–3 days.

For 3D cell spheroid culture, we created a new method to form and cultured cell spheroids with high throughput. Briefly, we prepared 0.8% agarose solution with agarose powder (50002, SeaKem) and double distilled water firstly, and then heated the solution in the microwave oven until it is completely dissolved. Next, we rapidly poured it into each hole of the culture plate in biosafety cabinet. Then we used silicon chips of different specifications made by nano optical lithography technology to prepare low-attachment microwell plates for cell spheroid culture. Finally, after 30 min irradiation with ultraviolet light of plates in biosafety cabinet, we injected cell suspension with proper density for high-throughput cell spheroid culture. The composition of culture medium and culture conditions are the same as 2D cells.

### Immunohistochemical and immunofluorescence staining

HCC tumor tissues were fixed in 4% PFA and embedded in paraffin, and then sectioned to 5 μm sections. For IHC, paraffin sections were rehydrated, incubated in antigen retrieval solution and blocking serum. The subsequent steps were performed using Vector kit (Vector Laboratories) according to the manufacturer‘s instructions. The IHC images were captured by the digital slide scanner (3D Histech).

For IF staining, the cells and spheroids were fixed in 4% PFA, permeabilized with 0.2% Triton X-100 and blocked with 10% Goat or Donkey serum and incubated with primary antibodies at 4°C overnight. They were then incubated with secondary antibodies for 1 h in the dark at room temperature, followed by 4′6-diamidino-2- phenylindole (DAPI) incubation for nuclear staining. The images of cultured cells, spheroids and organoids were taken by an Operetta High Content Imaging System (PerkinElmer).

### Quantitative real-time PCR (qRT-PCR) analysis

Total RNA was isolated from spheroids using a RNeasy mini kit (Qiagen). RNA was reverse-transcribed into cDNA using ReverTra Ace^®^ qPCR RT Master Mix (Toyobo) according to the manufacturer‘s in structions. qRT-PCR was performed on a Bio-Rad iQ5 Real-Time PCR detection system (Bio-Rad) with SYBR green master mix (Toyobo). Target gene expression levels were normalized to housekeeping gene β-actin by the 2 ^^ −ΔΔCt^ method. The primers used in this study are listed in Supplementary Table S4.

### Cell viability assay

For the monolayer cultured cells, the cell viability were detected using Cell Counting Kit-8 (CCK-8) (C0039, Beyotime) methods. After the drug treatment for 24 h, the cells were incubated with CCK-8 reagent for 1 h, and the OD value was measured using the microplate reader (BioTek, Synergy) at the wavelength of 450 nm. The cell viability of 3D spheroids were detected by Resazurin assay (R7017, Sigma), and the fluorescent intensity was measured at wavelengths of Ex530 nm/Em590 nm. All the test drugs were prepared with DMSO, and the cells treated with 0.1% DMSO were used as controls.

Additionally, the cell spheroids were also stained by LIVE/DEAD™ Viability/Cytotoxicity Kit (ThermoFisher Scientific). The spheroids were washed and stained with the Calcein AM and ethidium homodimer-1 (EthD-1), and confocal images of spheroids were acquired using an Operetta High-Content Imaging System (PerkinElmer), with a 20* Plan Fluor objective. A stack of 20 planes separated by 5 μm was acquired, starting at the well bottom and covering the lower half of each spheroid. All individual images were saved and used for automative quantitative analysis using Harmony^®^4.1 High-Content Imaging and Analysis Software.

### Cell apoptosis assay

Annexin V-FITC apoptosis detection kit (Beyotime) was used to detect the cell apoptosis and necrosis. Cells were seeded at a density of 2×10^5^ cells per well in a 6-well plate and cultivated overnight. The cells were then treated with the control or drug-containing media for 24 h. Subsequently, the cells were collected and stained with Annexin V-FITC and propidium iodide for 20 min and then analyzed using flow cytometry (Attune NxT, ThermoFisher Scientific).

### Western Blotting

After the drug treament at corresponding concentration for 24 h, the cells were harvested in lysis buffer (Beyotime Biotechnology) containing protease and phosphatase inhibitors (Roche). Lysates were sonicated for 30 s, and then spun at 12,000 rpm for 10 min at 4°C. Proteins were separated by 6–10% sodium dodecyl sulfatepolyacrylamide gel electrophoresis, transferred to polyvinylidene difluoride membranes, and probed with primary antibodies and horseradish-peroxidase (HRP)-conjugated anti-rabbit IgG antibodies. Target proteins were detected by enhanced chemiluminescence HRP substrate (Millipore).

### Statistical methods

Statistical analyses were performed using Graphpad Prism 8.4.0 software. Survival rates were calculated by the Kaplan-Meier method and compared using the log-rank test. Gene expression difference were compared by Wilcoxon rank sum test. IHC quantity results were analyzed by ImageJ software. The results of RT-PCR, IF, and cells/cell spheroids viability were analyzed using the unpaired independent sample *t*-test method. Combination Index (CI) were calculated by the formula: CI = D1/Dm1+D2/Dm2, where D1 and D2 are the concentrations of Drug one and Drug 2 with a certain level of cytotoxicity produced by combination drugs, and Dm1 and Dm2 are the concentrations of a single drug with the same effect respectively; The statistical experiments of this study were repeated more than 3 times. *p* values < 0.05 were considered statistically significant. *p-value < 0.05, **p-value < 0.01, ***p-value < 0.001, ****p-value < 0.0001.

## Results

### Expression and clinicopathological characteristics of FGF19-FGFR4-KLB axis in HCC

The FGF19-FGFR4 signaling axis has been linked to the development of various malignancies. Supplementary Figure S1A shows FGF19, FGFR4, and KLB expression in 31 cancers in the GEPIA-TCGA database. FGF19 is found to be low in most cancers; FGFR4 is highly expressed in many cancers, particularly in HCC and Intrahepatic Cholangiocarcinoma (ICC), with a significant difference in expression when compared to matched normal tissues; and KLB is found to be highly expressed in HCC patients’ cancer tissues and normal tissues adjacent to ICC patients.

**FIGURE 1:**
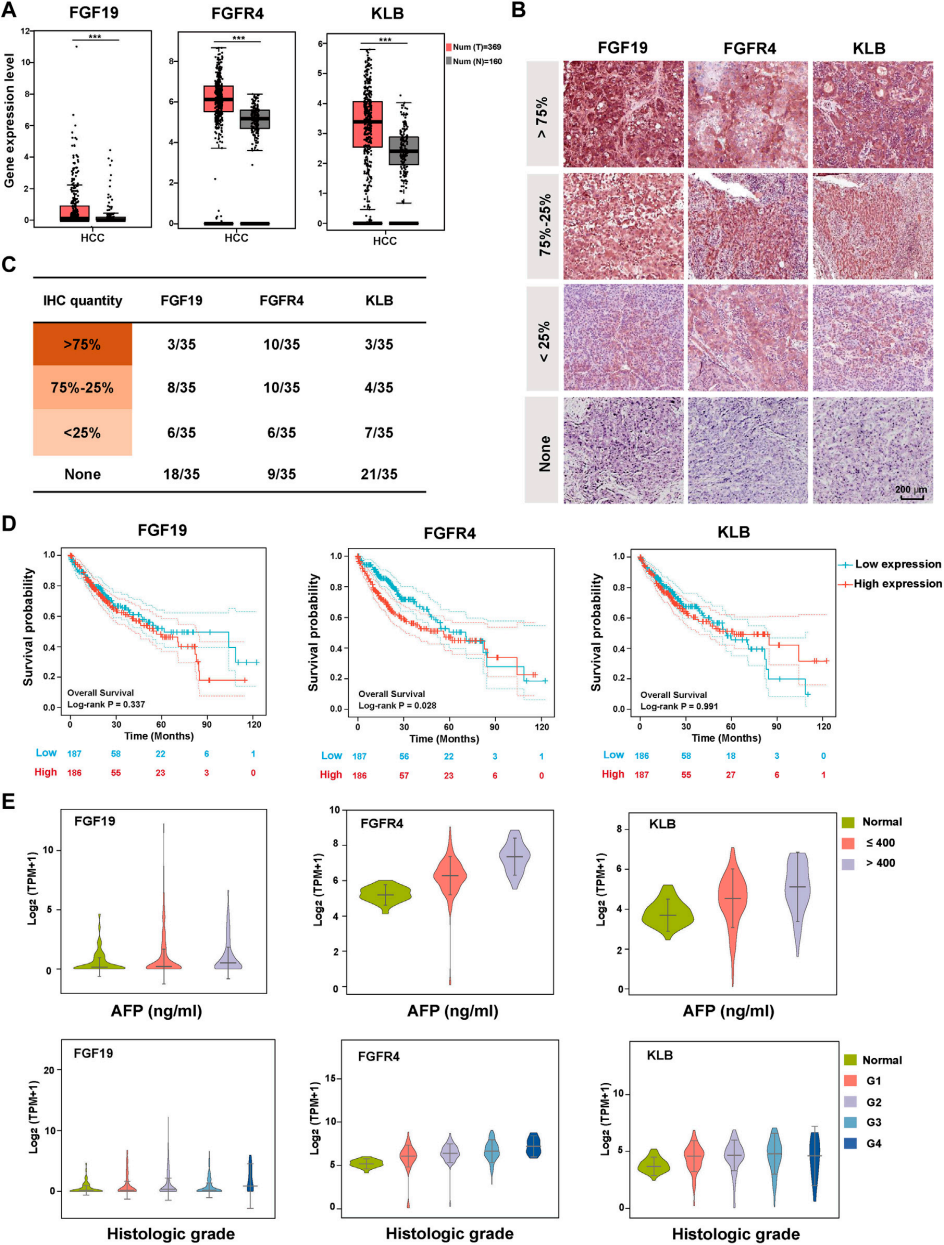
Expression and clinicopathological characteristics of FGF19-FGFR4-KLB axis in HCC. **(A)** The expression levels of FGF19, FGFR4, and KLB transcripts (TPM) in 369 cancer tissues (red) and 160 normal tissues (gray) of HCC patients. **(B)** Representative pictures showing the expression level of FGF19, FGFR4, and KLB protein evaluated by IHC in HCC tissues. **(C)** The statistical number of IHC FGF19, FGFR4, and KLB protein expression levels in HCC tissues. **(D)** The overall survival curve of HCC patients in the high and low expression groups of FGF19, FGFR4, and KLB, with the median as the cutoff point. **(E)** The relationship between the expression levels of FGF19, FGFR4, and KLB Log_2_ (TPM+1) and the clinicopathological characteristics (AFP level and Histological grade) of HCC in TCGA database. *p-value < 0.05, **p-value < 0.01, ***p-value < 0.001, ****p-value < 0.0001.

To determine which of FGF19-FGFR4-KLB axis can best represent the onset and progression of HCC, we investigated the GEPIA-TCGA database for their expression levels in HCC patients’ cancer tissues and normal tissues adjacent to cancer. All of FGF19, FGFR4, and KLB expression levels are greater in cancer tissues than in normal tissues next to malignancy (Figure 1A). Then we collected tissue samples of 10 HCC patients from clinical sources for transcript sequencing and found that their individual expression differences were consistent with the TCGA database, and it was obvious that FGFR4 had a significant and stable trend, which was more representative (Supplementary Figure S1B, S1C).

In addition to exploring the differences in gene expression of the three, we investigated differences in protein expression levels in HCC tissues. IHC assay was performed on the HCC tissues of 35 patients. According to the statistical results, FGFR4 was significantly higher than FGF19 and KLB in both the overall positive rate and the proportion of more than 75% amount, making it more representative (Figures 1B, C and Supplementary Table S5). We next looked through the Human Protein Atlas database for all the HCC-IHC data of FGF19, FGFR4, and KLB (8 cases each), and found that the FGFR4 quantity of 100% HCC samples reached 75%. In contrast, only six instances of KLB and 0 cases of FGF19 were reported. Supplementary Figure S1D displays the matching images. In general, FGFR4 is superior at foretelling the development of HCC.

We thoroughly investigated the relationship between FGF19-FGFR4-KLB and the clinical pathological information of HCC from the TCGA database, including Overall survival, AFP level, Histologic grade, Pathology stage, Metastasis stage, Tumor stage, Node stage, and Vascular invasion. When we analyze these data vertically, FGF19 does not significantly predict any of these indicators, and there is no correlation between the expression level and the grading or staging of these indicators (Figures 1D, E and Supplementary Figure S2). For FGFR4, there is a substantial positive link between the expression level and the patients’ overall survival, AFP level, Historical grade, Pathology stage and Metastasis stage. FGFR4 can not forecast trends for the markers of tumor stage, node stage, and vascular infection, but it can predict their occurrence. For KLB, the figure demonstrates that it has a strong correlation with AFP level and Metastasis stage in HCC patients. It can also foresee the occurrence of additional indications.

According to the findings of the previous two sections we can conclude that FGFR4 is the molecule in FGF19-FGFR4-KLB axis that is most closely associated with the formation and progression of HCC. It is also the most representative and predictive molecule.

### Exploration of the differences in patients with different FGFR4 expression

**FIGURE 2:**
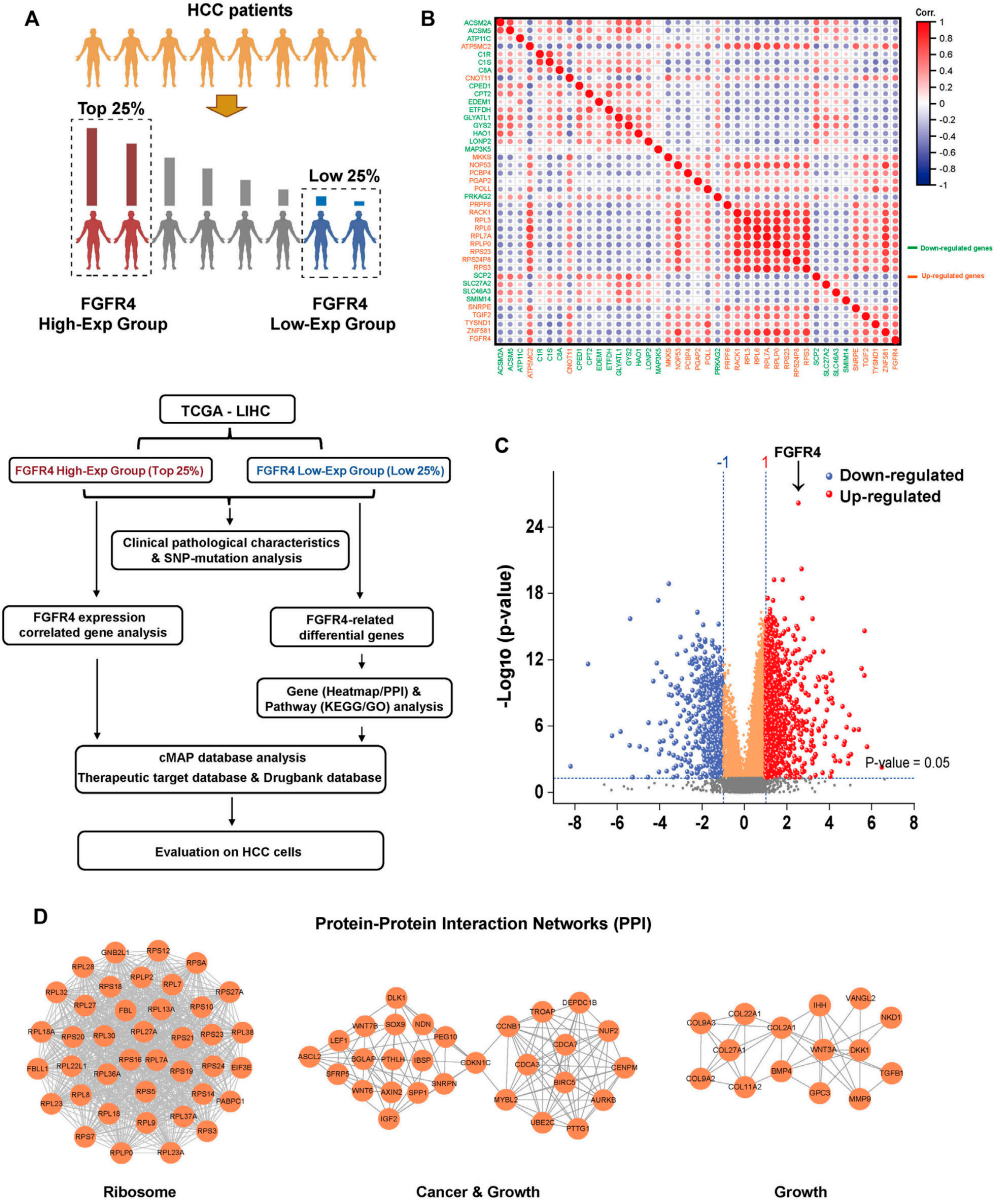
Genetic and pathway differences in populations associated with FGFR4 expression differences. **(A)** In TCGA data, HCC patients were grouped according to the expression of FGFR4 and the research process. **(B)** Correlation analysis of key top 20 genes expression in FGFR4 high or low expression group. **(C)** Differential gene volcano map based on FGFR4 high or low expression group. **(D)** The PPI network top three aggregations of up-regulated genes (*p* < 0.05, Log_2_ (Fold change) > 1) in the String database based on the FGFR4 high or low expression group.

To further explore the differences at the gene level among people with different FGFR4 expressions, we divided the HCC patients in TCGA database into two groups, according to FGFR4 gene expression (TPM) of the highest or lowest 25% (93 cases) for futher analysis, and we present the experimental design approach in Figure 2A. After grouping, there are still substantial disparities in the FGFR4, KLB, and AFP expression levels between the two groups, which clearly justifies the rationale of this grouping (Supplementary Figure S3A). We next explored the SNP (single nucleotide polymorphism) gene mutation type of the corresponding patients in the TCGA database. For HCC patients with high FGFR4 expression, 84.52% had mutations, with the first three mutations being Tumor Protein P53 (TP53) (35%), Catenin Beta 1 (CTNNB1) (21%), and Titin (TTN) (18%) (Supplementary Figure S3B), suggesting the predictive value of FGFR4 as a biomarker in HCC. Then, for correlation analysis, we choose the essential genes that express top 20 in the same direction (up or down-regulation) as FGFR4 expression for next analysis, as shown in Figure 2B.

To analyze the differential genes of the two groups, we discovered 970 up-regulated genes and 559 down-regulated genes after setting the *p*-value < 0.05, Log_2_ (FC) > + 1 or < -1 (Figure 2C). The heatmap was then utilized to demonstrate the difference in the expression of important genes between the FGFR4 High-Exp and the Low-Exp groups (Supplementary Figure S4A). Then we enriched and examined the KEGG and GO pathways of the two groups of differential genes. Many pathways show substantial changes between these two groups (Supplementary Figure S4B). These pathways include the Ribosome pathway, Metabolic pathway, Cellular process, and others (Supplementary Figure S4C, D and Supplementary Figure S5). Finally, we performed a Protein-Protein Interaction (PPI) network analysis for the up-regulated and down-regulated differential genes, and exhibited three key sets of PPI networks, which are respectively related to ribosome, cancer, growth, drug metabolism and cholesterol metabolism (Figure 2D and Supplementary Figure S4E).

Based on the results of the above sections, we established that FGFR4 is the most significant to the clinicopathological aspects of HCC. Then, we divided HCC patients in the TCGA database into High-Exp and Low-Exp groups based on FGFR4 gene expression levels, thoroughly investigated the gene difference between the two groups, re-evaluated the value of FGFR4 in predicting the occurrence and development of HCC, as well as patient prognosis, and revealed some of the pathway mechanisms.

### Drug screening for improving the effect of FGFR4 inhibitors

**FIGURE 3:**
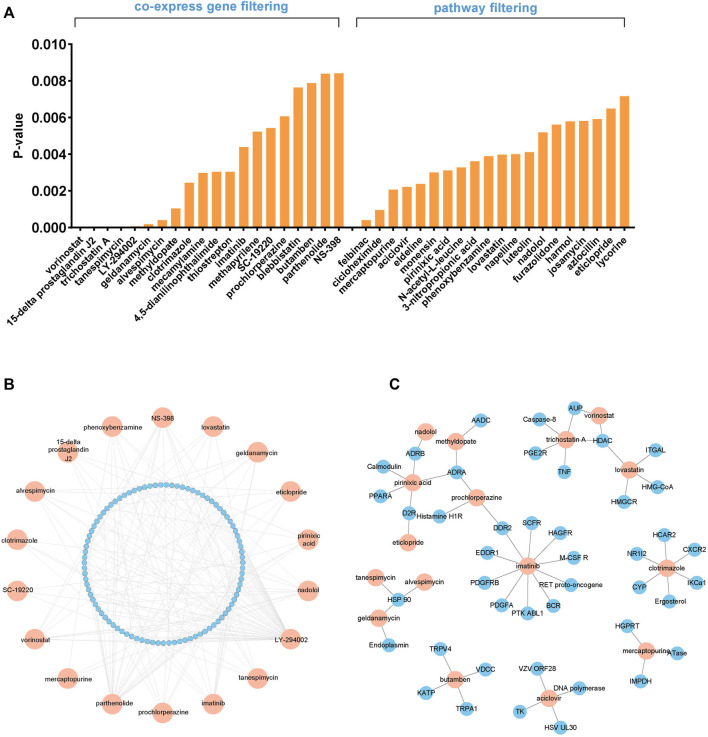
Screening of the potential FGFR4 inhibitor combinations. **(A)** The cMap database screened 40 potential combination drugs based on FGFR4 co-expression genes and enrichment pathways. **(B)** The TTD explores the pathways of candidate drugs. **(C)** The Drugbank database explores the targets of candidate drugs.

As was already noted, several FGFR4 inhibitors have been developed and have begun to be employed in clinical trials, but the overall results have not been promising, making it crucial to explore the potential drugs that could be combined with FGFR4 inhibitors. We first predicted the possible drugs through the cMap database. The cMap, created by Lamb et al., in 2006, is a large public database that contains drugs and gene signatures, and illustrates the relationships between genes, medications, and disorders. In order to find a list of small compounds with the potential to cure HCC, a query was made using the intersected genes from the aforementioned analysis, including co-expression genes and enrichment pathways. We selected the first 20 drugs respectively for further research, according to the *p*-value (Figure 3A and Supplementary Table S6, 7). The TTD was used to test these 40 medicines’ pathways, and the Drugbank database was used to screen the targets (Figures 3B,C). The following seven drugs were identified using the data from the three databases: Geldanamycin, Imatinib, LY-294002, Parthenolide, Tanespimecin, Trichostatin A, and Vorinostat. Specific drug information has shown in Supplementary Table S2 and Supplementary Table S8.

### Evaluation of FGFR4 inhibitors combinations in HCC cells

**FIGURE 4:**
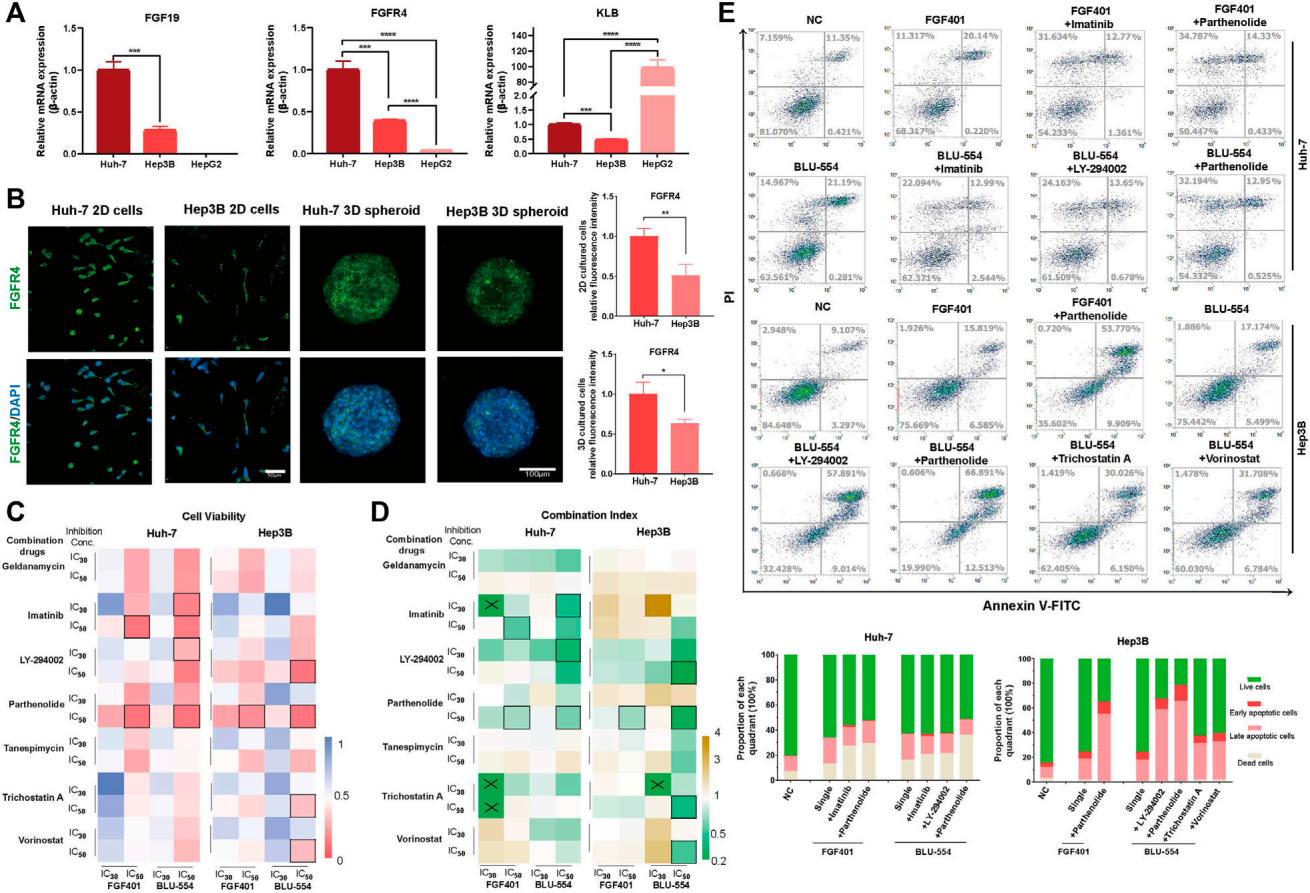
Evaluation of FGFR4 inhibitors combinations in HCC cells. **(A)** The expression of FGF19, FGFR4, and KLB in Huh-7, Hep3B and HepG2 cells was detected by qPCR. **(B)** IF staining to detect the FGFR4 protein level in Huh-7 and Hep3B cells. **(C)** Huh-7 and Hep3B cells were treated with two FGFR4 inhibitors (FGF401 and BLU-554) and seven candidate drugs. CCK-8 assay was used to quantify the cell activity. **(D)** The drug combination index is calculated according to the cell activity and drug concentration and is displayed in the heatmap. “×” indicates that the CI cannot be calculated, and “□” indicates the selected combination drug schemes. **(E)** The flow cytometry apoptosis detection and statistical results of each quarter in each of five combination drug schemes and control groups in Huh-7 and Hep3B cell lines. *p-value < 0.05, **p-value < 0.01, ***p-value < 0.001, ****p-value < 0.0001.

We selected three HCC cell lines (Huh-7, Hep3B and HepG2) to detect the FGF19, FGFR4, and KLB expression levels. In contrast to Huh-7 and Hep3B, HepG2 cells do not express FGF19, and FGFR4 expression is only moderately expressed, as shown in Figure 4A. Therefore, for further investigation, we chose Huh-7 and Hep3B cell lines. We performed the IF staining to check the FGFR4 expression on protein level, and found that Huh-7 cells expressed FGFR4 at a greater level than Hep3B cells in both monolayer and 3D spheroid cultures (Figure 4B).

Next, we treated Huh-7 and Hep3B cells with two FGFR4 inhibitors (FGF401 and BLU-554) as well as seven candidate single drugs, drawing the cell viability curves of each of these nine single drugs using the CCK-8 assay (Supplementary Figure S6A). We chose the 30% and 50% of inhibition concentration (IC_30_ and IC_50_) of these above nine drugs to investigate their potential combinations (Supplementary Figure S6B and Supplementary Table S9). The heatmap in Figure 4C shows the effect of combined usage of drugs (Supplementary Table S10). After that, we calculated the combined drug index (CI), and showed the result in Figure 4D and Supplementary Table S11. If the CI is less than 1, the two drugs are synergistic, and if it is greater than 1, they are antagonistic. For further evaluation of cell apoptosis and necrosis on HCC cells, we selected a few schemes using the criterion of cell viability (CV) < 0.1 or CI < 0.6.

The results demonstrated that the combination effect was significantly improved compared to that of a single treatment, and we first determined that Parthenolide was the most often combined drug with FGFR4 inhibitors both FGF401 and BLU-554 (Figure 4E).

### Evaluation of the combined schemes effect of on 3D cultured HCC spheroids

**FIGURE 5:**
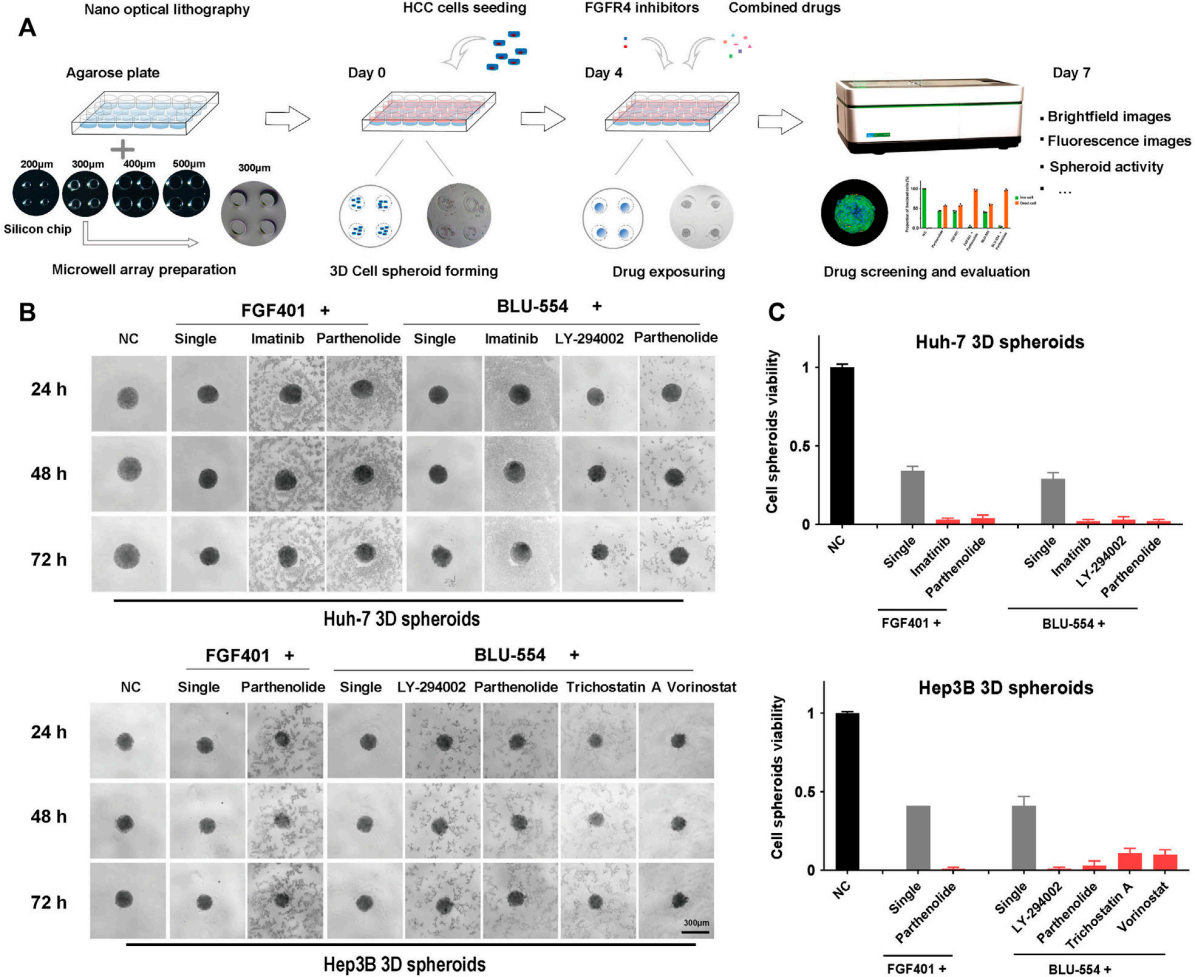
3D cell spheroids to evaluate the effect of combined schemes. **(A)** Schematic diagram of agarose microwell plate fabrication and cell spheroid culture process. **(B)** The state of Huh-7 and Hep3B 3D cell spheroids using the combination drug schemes were photographed in the bright field under the microscope. **(C)** The activity of Huh-7 and Hep3B 3D cell spheroids using the combination drug schemes was detected by the Resazurin assay.

In order to better simulate the microenvironment of solid tumors *in vivo* and more realistically reflect the effect of drugs, 3D cell spheroids were used to verify the above combined schemes. We used nano optical lithography technology to make the silicon chips, which is used to make agarose microporous culture plate for high-throughput generating cell spheroids, as shown in Figure 5A. After testing the pore size of 200 μm, 300 μm, 400 μm, and 500 μm respectively, we selected the pore size of 300 μm for cell spheroid formation. After 3 days, these HCC spheroids were treated with the above drug combinations for further evaluation. We used a microscope to continuously take images of cell death and shedding of 3D cell spheroids during drug exposure. The cell abscission in the cell spheroids treated with the combination of drugs is evident in Figure 5B, but the volume of the cell spheroids has not altered much compared to the group receiving only one treatment, which may be a result of the loose intercellular connections. The Resazurin assay was used to evaluate the cell activity of the tumor spheroids. As shown in Figure 5C, it is easy to see from the above results that the effects of the combination of FGFR4 inhibitors and Parthenolide were obvious both on Huh-7 and Hep3B spheroids. Parthenolide might be used as a potential combination strategy.

**FIGURE 6:**
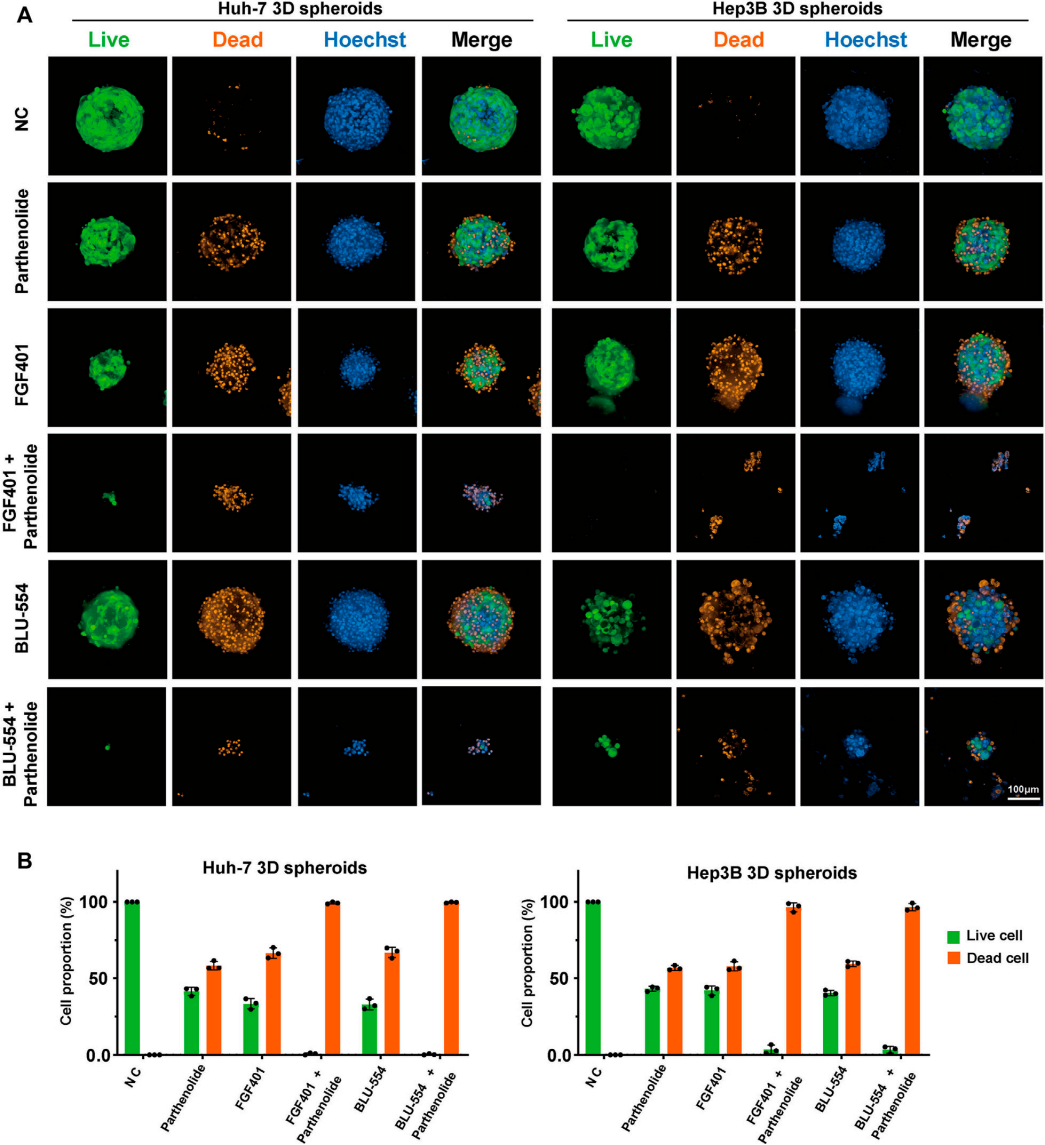
3D cell spheroids to evaluate the effect of combined application of FGFR4 inhibitors and Parthenolide. **(A)** The Live/dead probe staining was used to detect the living or dead levels of 3D cell spheroids using the combination drug schemes. **(B)** The Live/dead probe staining was used to detect the living or dead levels of 3D cell spheroids using the combination of drug schemes.

To further evaluate the possible potential combinations, high content imaging and analysis were performed to study the percent of living and dead cells in drug treated spheroids. After staining, the spheroids, treated with one or another combined drugs, were used to generate high-resolution images by a spinning-disk confocal using a Z-stepping model. With respect to multiparameters, we visually and automatically analyzed the drug-induced alterations in Huh-7 and Hep3B spheroids. The results were presented in Figure 6A. The Live/dead probes were used to staining the cells at the endpoint of incubation time, counted the number of living cells and total number of cells, and calculated the proportion of living and dead cells to evaluate the drug effect. The data demonstrate that the combination treatment has a considerable effect (Figure 6B). At the same time, we tested single drug effect of Parthenolide by the microscope bright field observation and Resazurin assay on 3D cell spheroids to compare the effects of combined application of FGFR4 inhibitors and Parthenolide (Supplementary Figure S7). And we also evaluated the other combinations, and showed the results of Live/dead assay in Supplementary Figure S8.

### Exploration of the mechanism of FGFR4 inhibitors and Parthenolide combined application

**FIGURE 7:**
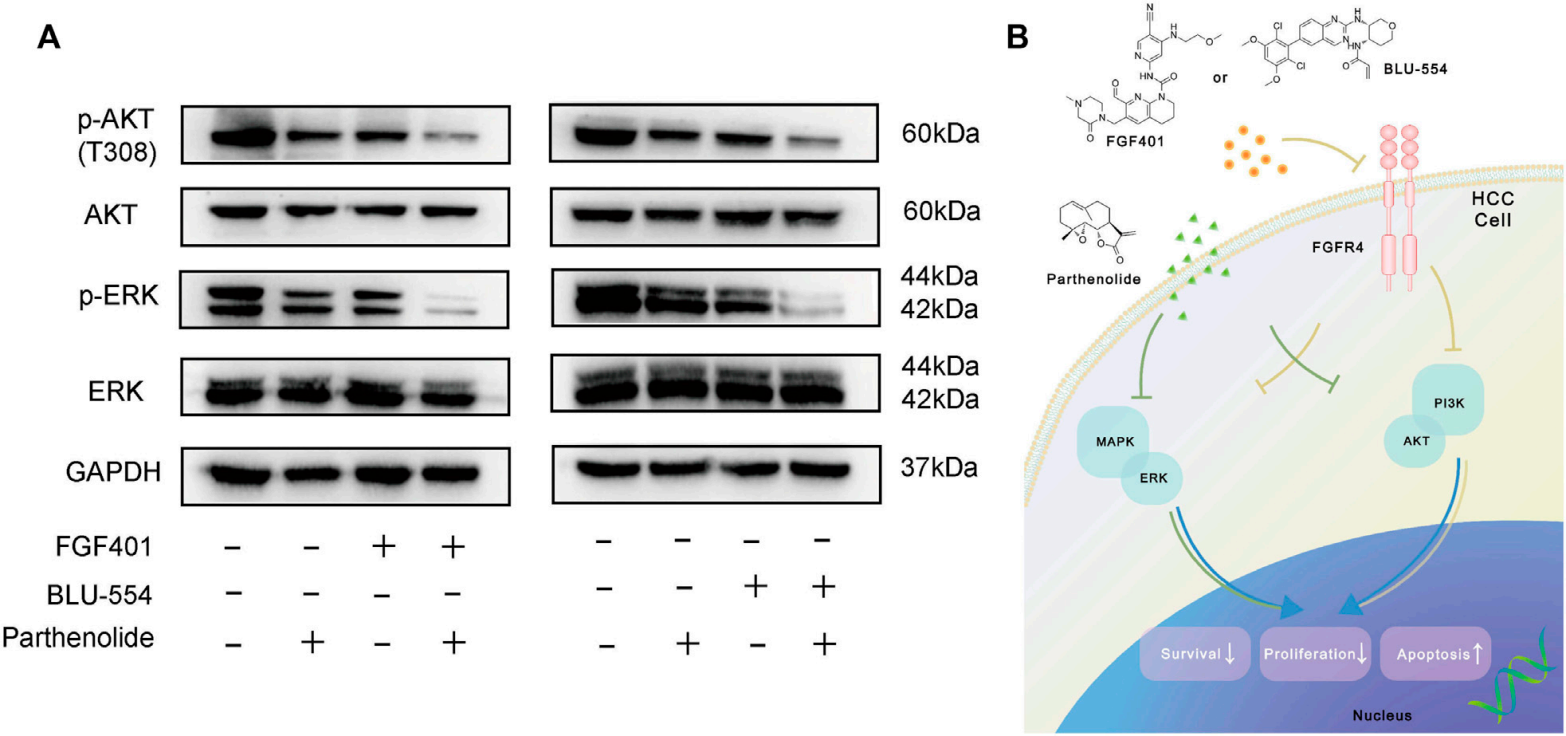
Mechanism exploration of combined schemes. **(A)** WB assay was used to examine the mechanism of the combined schemes, Huh-7 cells were treated with drugs for 12 h. **(B)** The mechanism pathway model diagram of the combination drug schemes.

Finally, we tried to interpret the mechanism of the combined scheme of FGFR4 inhibitors and Parthenolide. Figure 7A showed that the levels of p-AKT and p-ERK were significantly decreased by FGF401 or BLU-554 in combination with Parthenolide compared with single drug or no drug effects. And we speculated the increased effect of the combined scheme might be attributed to the synergistic suppression of the MAPK-ERK and PI3K-AKT pathways, which regulate HCC cell survival and proliferation (Figure 7B). Based on the above results, we identified that the combining FGFR4 inhibitors with Parthenolide is a successful combination scheme with promising therapeutic implications.

## Discussion and conclusion

The research and development of anti-tumor drugs not only depends on the discovery of new targets, but also the high-fidelity model used for drug screening and assessment is important. 3D cell culture offered more closely mimic some features of solid tuners, and we have developed a new high-throughput method for preparing cell spheroids, which makes more accurate drug evaluation possible. To find the potential drugs for FGFR4-specific HCC, we divided the HCC patients from TCGA were into two groups using the FGFR4 expression. FGFR4 and AFP were definitely significantly different between the two groups. In the high expression group, TP53 mutation occurred in 35% of patients, and CTNNB1 mutation occurred in 21% of patients. We reviewed the relevance and significance FGFR4, and targeting FGFR4 will be an effective and important means for HCC patients.

However, this is confronted with difficulties at development of FGFR4-specific inhibitors. To overcome the problem of these inhibitors’ limited efficacy, we have sought to explicitly identify drugs for FGFR4 high expression HCC using the specific gene expression signatures. Using gene data features to predict potential therapeutic drugs or drug combinations is an area of great concern at present, which can greatly reduce experimental costs and achieve high-throughput and high matching drug screening. For the drug combination screening, there are several deep learning-based methods including MatchMaker, DeepSynergy, EC-DFR and so on. DeepSynergy is the first method to predict drug combination synergy based on deep learning. Compared with other methods, DeepSynergy uses a wider range of data, integrating chemical and genomic information as input data. Because of the integration of data from different sources, the model also adopts a standardization strategy to explain the heterogeneity of input data (Preuer K. et al., 2018). cMap is a gene expression database, which detects the gene expression differences after drugs (including small molecules) have processed human cells, and establishes a biological application database that is related to drugs, gene expression and diseases. It can help researchers quickly use gene expression profile data to compare drugs highly related to diseases in the field of drug research and development, and the possible mechanism of action of drug molecules can be summarized (Corsello SM. et al., 2017). Several previous studies have used HCC gene signatures to the cMap database, which contains gene expression profiles from five non-HCC human cancer cell lines treated with 1309 chemicals (Lamb J. et al., 2006). Two studies queried against the cMap database using HCC gene expression signatures, and validated several drug candidates *in vitro* and *in vivo* (Woo et al., 2009; Chen et al., 2011). Using the connectivity mapping predictions and further validations by pathways and targets, we screened seven drugs that maybe effectively combined with FGFR4 inhibitors. Then, we combined two FGFR4 inhibitors (FGF401, BLU-554) with seven candidate drugs (Geldanamycin, Imatinib, LY-294002, Parthenolide, Tanespimycin, Trichostatin A and Vorinostat), and determined that the combination of FGFR4 inhibitors (FGF401, BLU-554) and Parthenolide had the best effect through experimental exploration of 2D cells and 3D cell spheroids.

Parthenolide is a naturally occurring biological aminobenzoic acid. It is an NF- κB inhibitor, that inhibits histone deacetylase 1 (HDAC-1) and DNA methyltransferase 1 (DMT-1) (Bork et al., 1997; Freund et al., 2020). It has also been found to have anti-cancer activity in a variety of tumors, such as breast tumors, colorectal cancer, renal cell carcinoma, and et al. (Araújo et al., 2020; Liu D. et al., 2021; Liu X. et al., 2021). Combination treatment with Parthenolide and 5-FU provides synergistic anti-cancer effects *in vitro* and *in vivo* (Kim S.-L. et al., 2013; Liu Dajun et al., 2013; Ding et al., 2019). *Pan* Liang et al. found that Sorafenib and Parthenolide showed high-quality synergistic intracellular uptake, cell proliferation inhibition, and migration inhibition of HCC *in vitro*. At the same time, *in vivo* anti-tumor studies demonstrate that synergistic drugs showed a higher tumor inhibition rate than single drugs (Liang et al., 2020).

Since we have skilled high-throughput cell spheroid culture and assessment procedures, we confirmed the effect of the combination application of FGFR4 inhibitor and Parthenolide on not only monolayer cultivated cells but also 3D cultured spheroid. Previous publications have demonstrated that growing cancer cells in the form of 3D tumor spheroids can be more predictive of the *in vivo* study outcomes compared to the 2D cell culture method. We have studied the size-dependent localization and penetration of gold nanoparticles in tumor using the multicellular spheroids culture system (Huang K. et al., 2012; Huo S. et al., 2013). Although 3D cell spheroids have been widely considered as an excellent drug screening model, they still face the dilemma of high-throughput 3D cell spheroid culture until now. The formation and culture of 3D cell spheroids involved various methods, such as hanging drops (Tung Y.C. et al., 2011), non-adjacent surfaces (Napolitano A.P. et al., 2007), micro/nanostructures (Yoshii Y. et al., 2015) and so on. However, it seems that these methods require complex processes, time-consuming and low production, in addition, the volume of 3D cell spheroid produced is not equal, which makes it difficult to form large-scale. When seeking to design an efficient 3D cell spheroids culture system, we aimed to create a microwell platform that can quickly form large uniform sized micro tumor tissues. The uniform volume of each micro tumor tissue benefits from the assembly mode of the platform, which can accurately control the number of cells. In the 3D cell spheroids experiment, the combined treatment group has good effects on the activity detection, bright field observation and Live/dead staining. At the same time, we also observed that the Huh-7 cell line is more sensitive to FGFR4 inhibitors than Hep3B cells, possibly due to the abundance of FGFR4 receptors in this cell line. This conclusion can be inferred from the difference in Flow cytometry and 2D or 3D cell activity detection.

Interestingly, we discovered that 3D cell spheroid is more responsive to FGFR4 inhibitors and related medicines than monolayer 2D cells after extensive drug screening and morphological and activity evaluation. The first reason might be that the medications mentioned above have a high tumor permeability. The second explanation might be that when 3D solid cell spheroid develops a close connection, it forms a completely oxidized outer layer and anoxic center. However, several receptors triggered by complete oxidation and hypoxia, such as Acyl Coenzyme A Oxidase 1 (ACOX1) (Chen X. et al., 2018) and hypoxia inducible factor-1 (HIF-1) (Li Q. et al., 2021), have interaction with FGFR4, improving the sensitivity of 3D cell spheroid to these treatments. The particular mechanism requires more investigation and interpretation.

In conclusion, 3D cell spheroids culture provide a promising disease models that can not only help us better understand disease biology, but may also be used to precisely build target tissue models for screening medications or evaluating therapeutic effects *in vitro*. Through the screening and evaluation of valuable cell spheroids with high content imaging, we are convinced that the combination of FGFR4 inhibitor and Parthenolide provides a new and valuable strategy for the treatment of FGFR4 positive HCC patients, which enables great potential applications in clinic.

## Data Availability

The datasets presented in this study can be found in online repositories. The names of the repository/repositories and accession number(s) can be found in the article/Supplementary Material.
