# Supplementary material for: Generation of multicellular tumor spheroids with micro-well array for anticancer drug combination screening based on a valuable biomarker of hepatocellular carcinoma
Source: Front Bioeng Biotechnol. 2022 Dec 1;10:1087656. doi: 10.3389/fbioe.2022.1087656 (PMC9751422; doi:10.3389/fbioe.2022.1087656)
Supplement: Supplementary file 1 [file DataSheet1.docx]

Supplementary Material


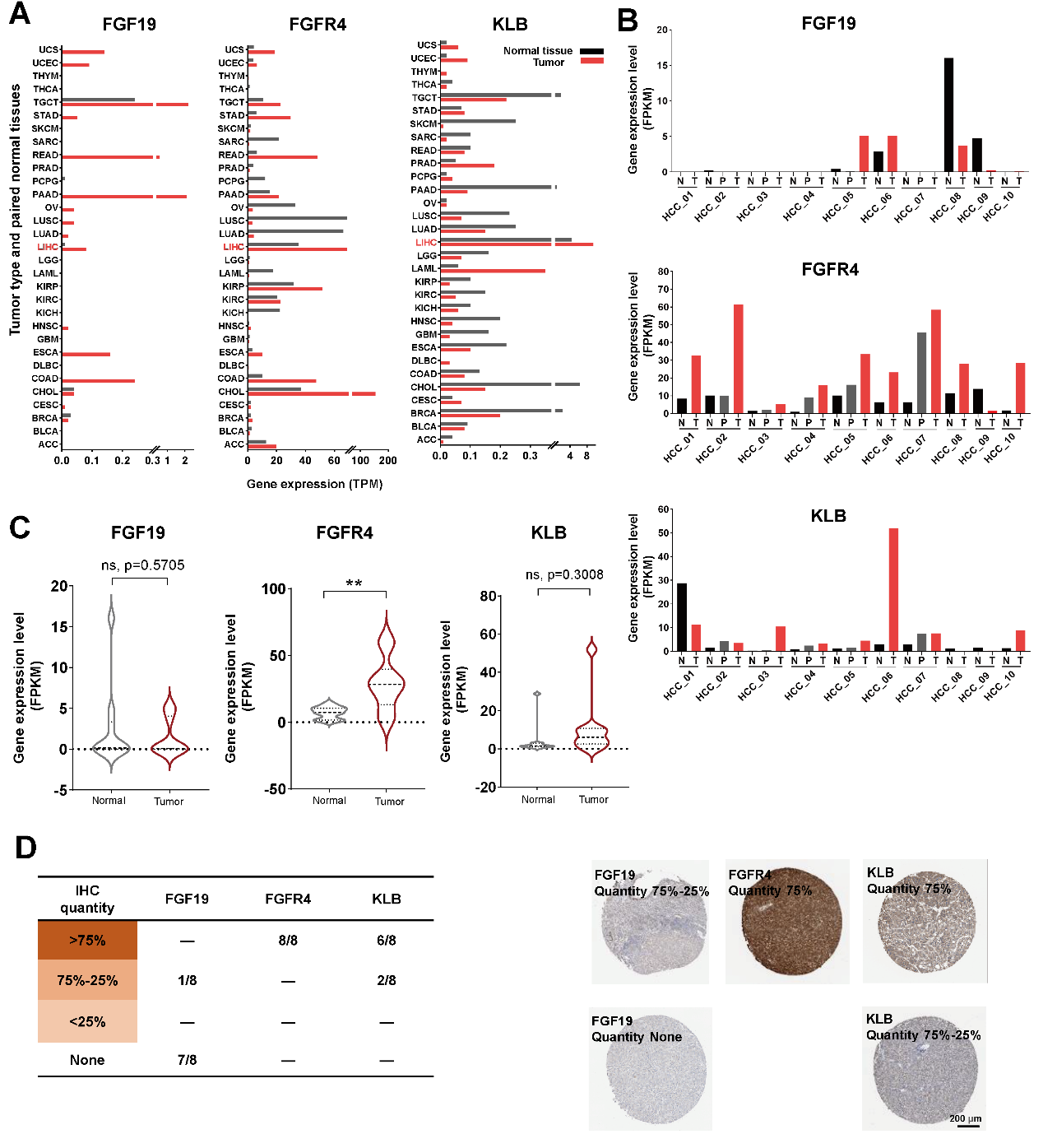


**FIGURE S1.** (A) The expression levels of FGF19, FGFR4, and KLB transcripts (TPM) in cancer tissues and corresponding normal tissues of 31 cancers in the GEPIA-TCGA database. (B, C) The expression levels of FGF19, FGFR4, and KLB transcripts (FPKM) in 10 HCC patients' cancer tissues (T), adjacent tissues (P), or normal distant cancer tissues (N). (D) IHC information of the expression level of FGF19, FGFR4 and KLB proteins in HCC tissues in the Human Protein Atlas database. * P value<0.05, ** P value<0.01, *** P value<0.001, **** P value<0.0001


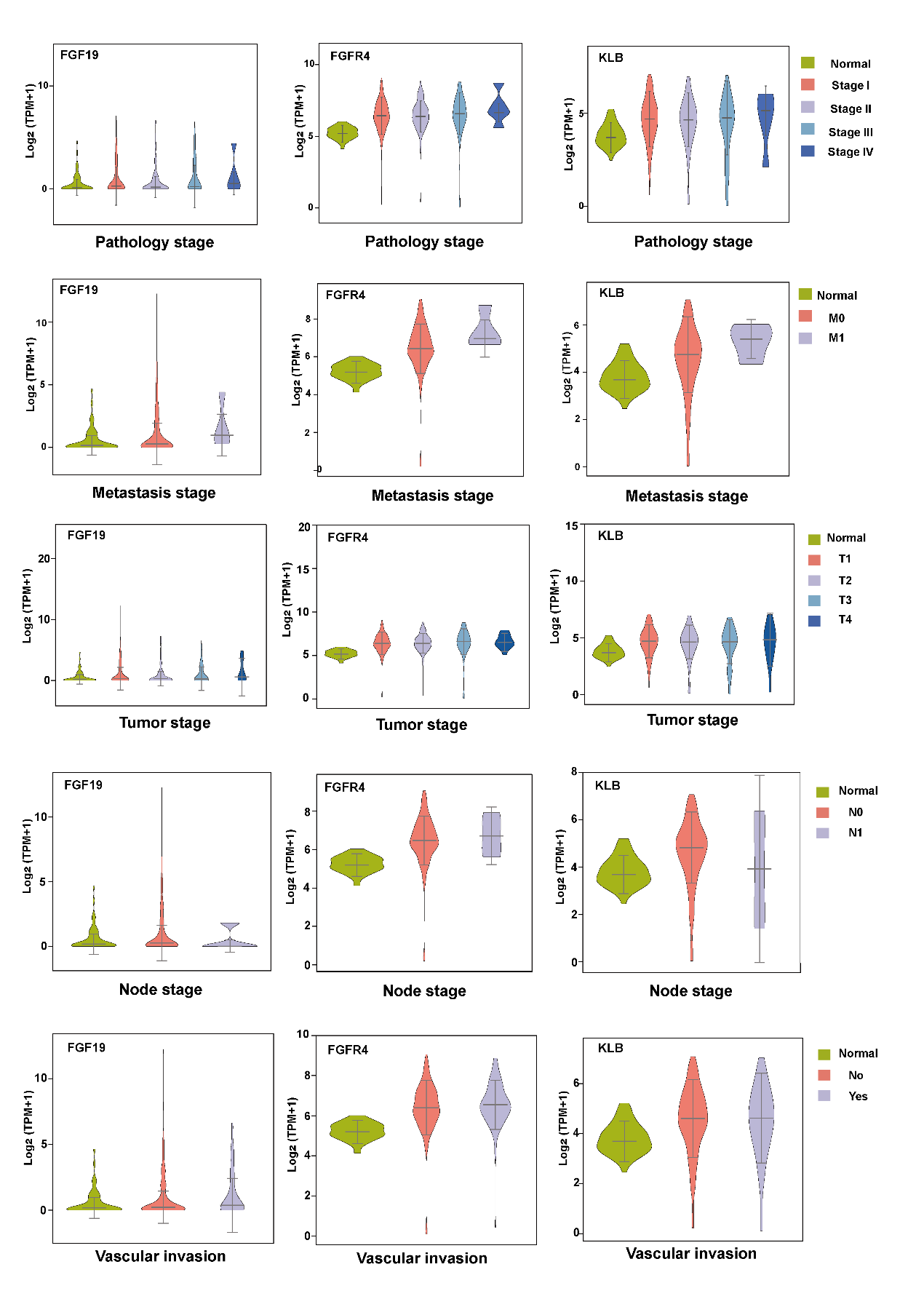


**FIGURE S2.** The relationship between the expression levels of FGF19, FGFR4, and KLB Log_2_ (TPM+1) and the clinicopathological characteristics (Pathological stage, Metastasis stage, Tumor stage, Node stage and Vascular invasion) of HCC in TCGA database.


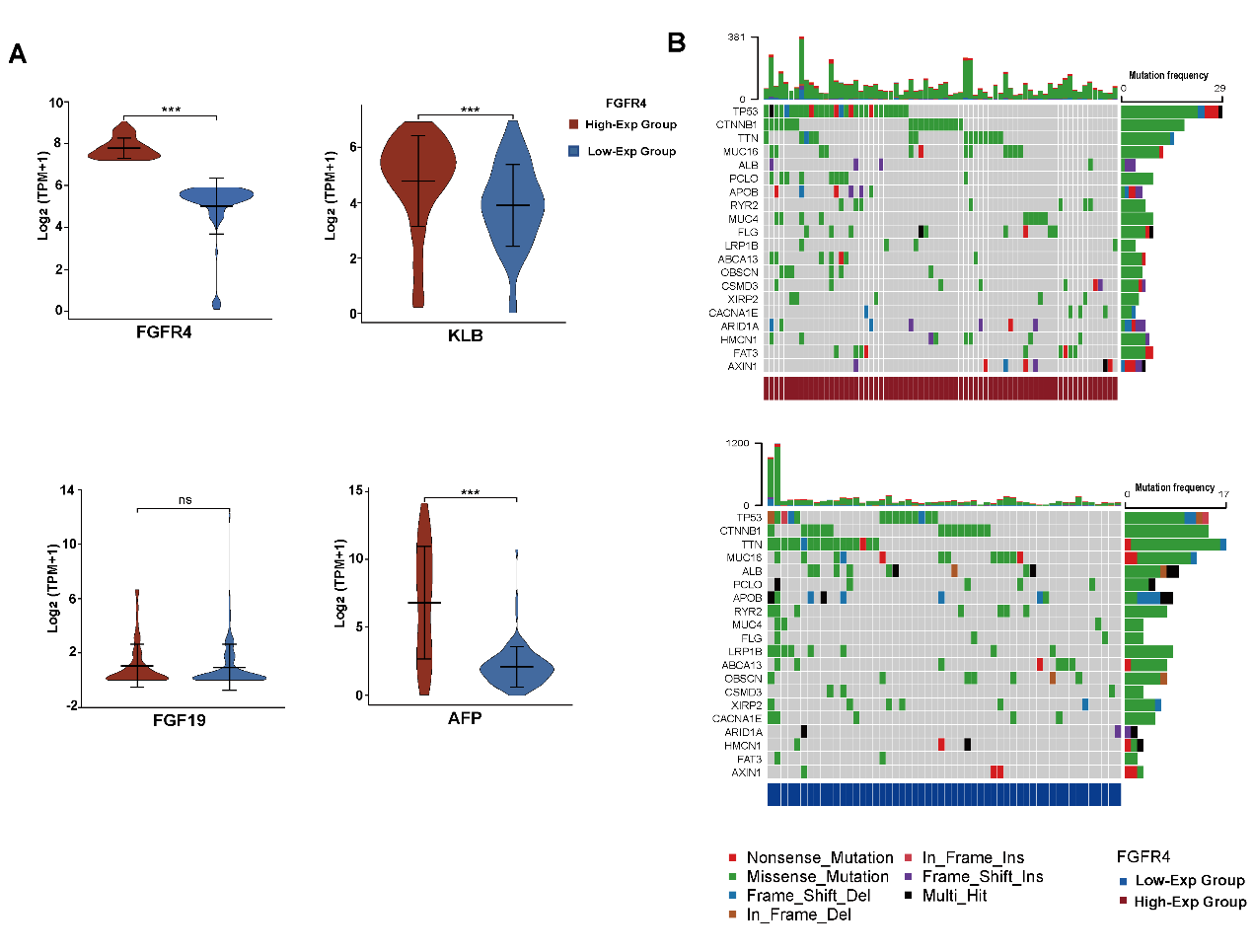


**FIGURE S3.** (A) The expression levels of FGFR4, KLB, FGF19, and AFP in HCC patients group with high or low expression of FGFR4 in the TCGA database (Log_2_ (TPM+1)). (B) Mutation type analysis of SNP (single nucleotide polymorphism) gene in HCC patients with high or low expression of FGFR4. * P value<0.05, ** P value<0.01, *** P value<0.001, **** P value<0.0001


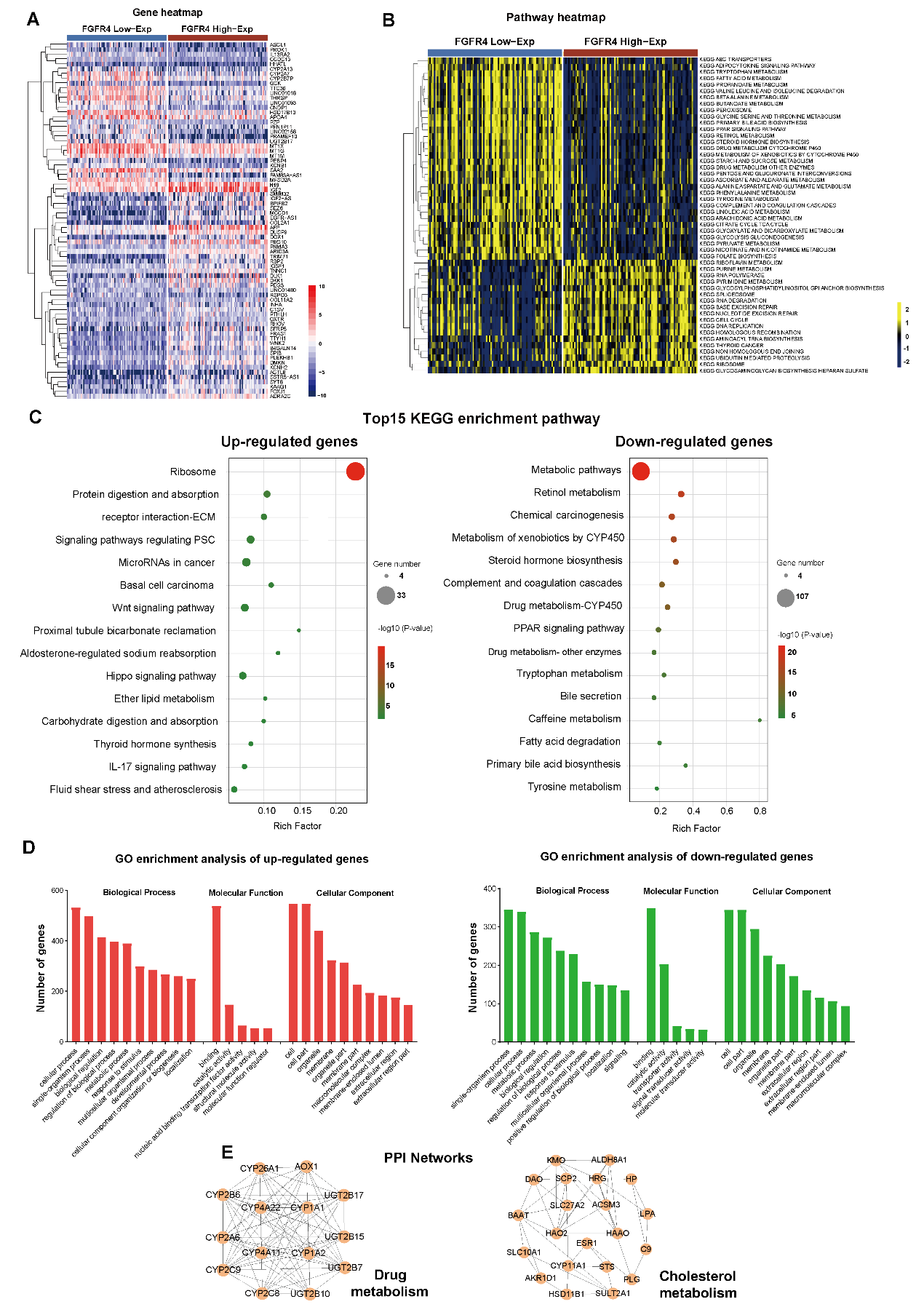


**FIGURE S4.** (A) Key genes heat map based on FGFR4 high or low expression group. (B) Key KEGG pathways heat map based on FGFR4 high or low expression group. (C) Enrichment analysis of KEGG pathway of up-regulated genes (P<0.05, Log_2_ (Fold change) >1) and down-regulated genes (P<0.05, Log_2_ (Fold change) <-1) based on FGFR4 high or low expression group. (D) The GO pathway enrichment analysis of up-regulated (P<0.05, Log_2_ (Fold change)>1) and down-regulated genes (P<0.05, Log_2_ (Fold change) <-1) based on the FGFR4 high or low expression group. (E) The PPI network aggregations of down-regulated genes (P<0.05, Log_2_ (Fold change) <-1) in the String database based on the FGFR4 high or low expression group.


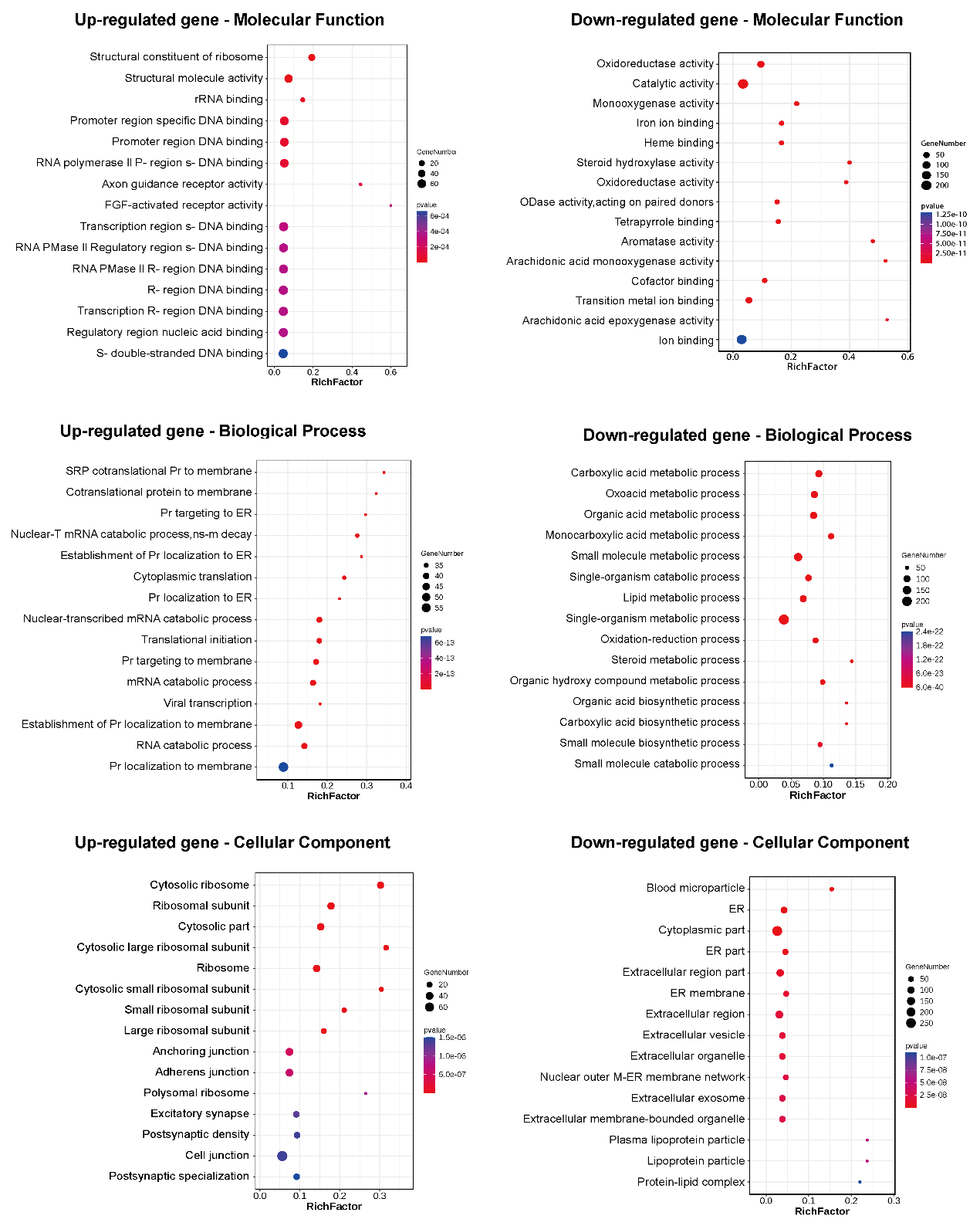


**FIGURE S5.** The GO pathway enrichment analysis of up-regulated (P<0.05, Log_2_ (Fold change)>1) and down-regulated genes (P<0.05, Log_2_ (Fold change) <-1) based on the FGFR4 High-Exp or Low-Exp group.


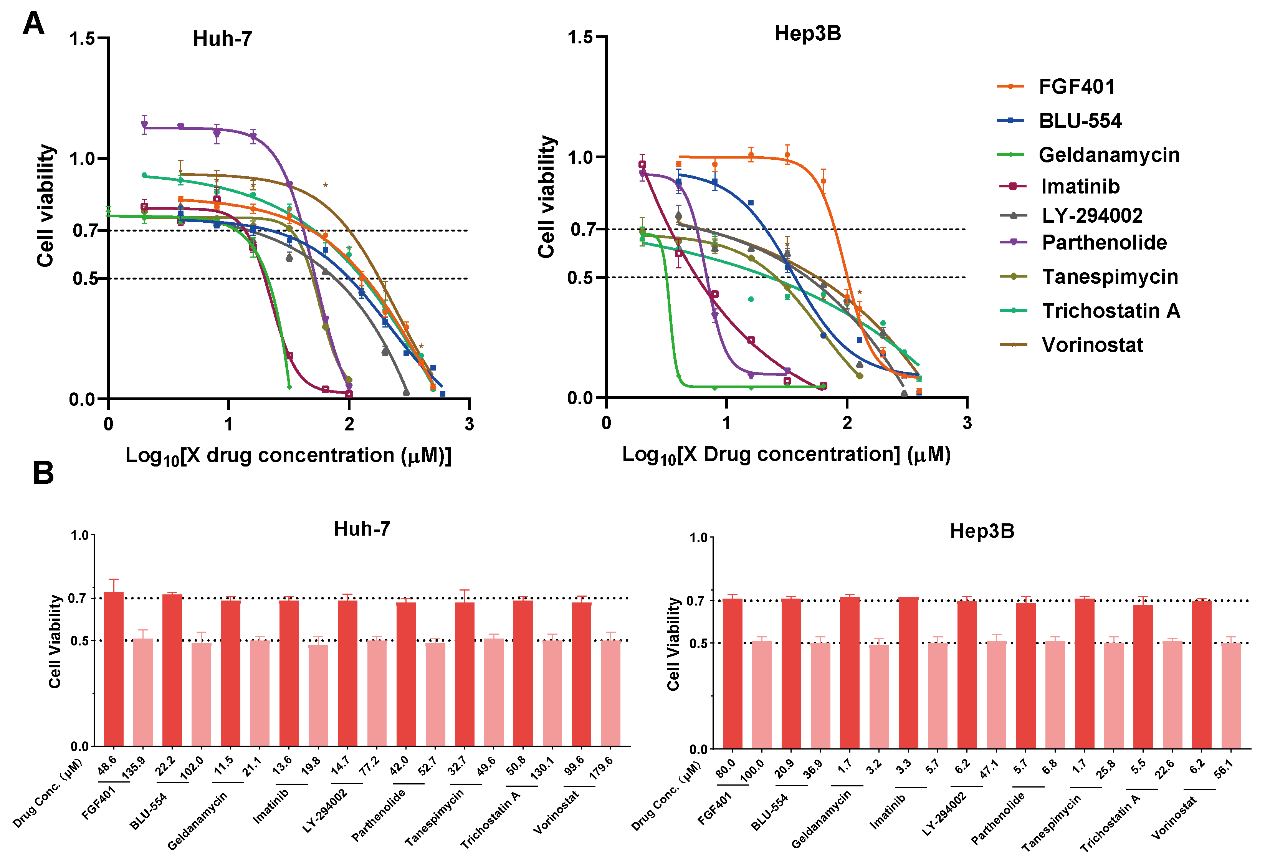


**FIGURE S6**. (A) The CCK-8 assay detected the cell viability curves of FGFR4 inhibitors (FGF401 and BLU-554) and 7 candidate combination drugs. (B) The IC_30_ and IC_50_ of 9 drugs were verified by CCK-8 assay, which calculated according to the cell viability curves.


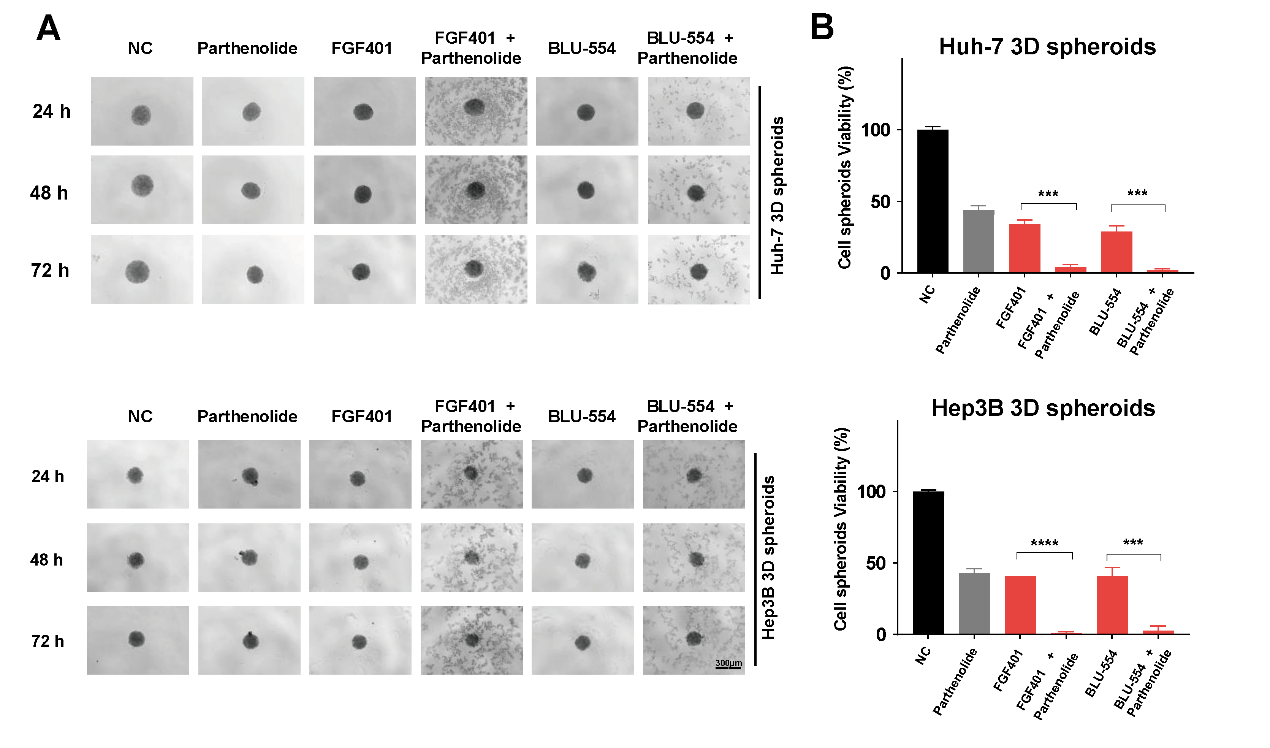


**FIGURE S7.** (A) The state of Huh-7 and Hep3B 3D cell spheroids using the combination drug schemes were photographed in the bright field under the microscope. (B) The activity of Huh-7 and Hep3B 3D cell spheroids using the combination drug schemes was detected by the Resazurin assay. * P value<0.05, ** P value<0.01, *** P value<0.001, **** P value<0.0001


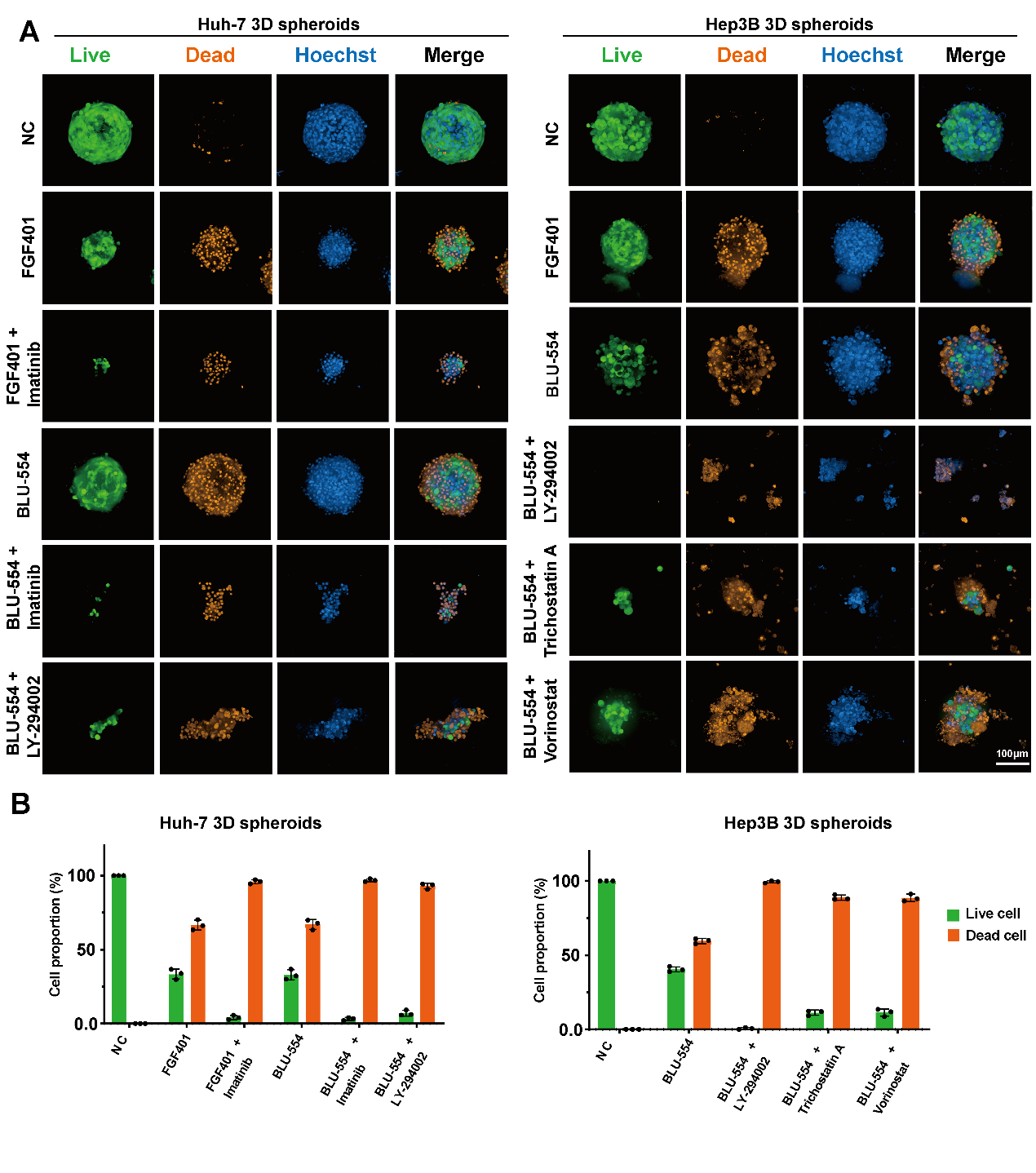


**FIGURE S8.** (A) The Live/dead probe staining was used to detect the living or dead levels of 3D cell spheroids using the combination drug schemes. (B) The Live/dead probe staining was used to detect the living or dead levels of 3D cell spheroids using the combined schemes.

**TABLE S1. The summary of FGFR4 inhibitors.**

| No. | Drug name | CAS No. | Structure | Company | Indication disease | drug delivery route | Research stage | Clinical Trials number | Clinical Trials Status | Latest research progress |
| --- | --- | --- | --- | --- | --- | --- | --- | --- | --- | --- |
| 1 | FGF401 (Roblitinib) | 1708971-55-4 | 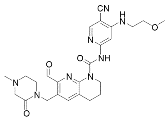 | Novartis | HCC | PO | Phase II | NCT02325739 | Recruitment Status: Completed;  First Posted: December 25, 2014;  Results First Posted: December 17, 2020;  Last Update Posted: December 17, 2020. | The research results published by the researchers in the clinicaltrial database show that among the 11 groups of patients participating in this clinical experiment, 3.8% of the patients in one group reached the “complete response”, which is already the highest proportion; For “partial response”, this proportion is up to 20%; For “stable disease”, the highest proportion is 57.1%; For more than half of the patients in most groups, HCC is still progressing. |
| 2 | BLU-554  (Fisogatinib) | 1707289-21-1 | 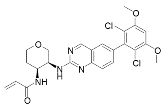 | Blueprint Medicines | HCC | PO | Phase I | NCT02508467 | Recruitment Status: Active, not recruiting;  First Posted: July 27, 2015;  Last Update Posted: January 11, 2022. | According to the phase I preliminary clinical data published by blueprint medicines at the 2017 European Society of Medical Oncology Congress (ESMO): as of August 18, 2017, among the 38 FGFR4 driven HCC patients who had undergone multiple chemotherapy, 6 patients (16%) achieved objective remission, 26 patients (68%) achieved disease control, and 18 patients (49%) had reduced tumor burden (Kim et al. 2017). |
|  |  |  |  |  | Locally Advanced or Metastatic HCC | PO | Phase Ib/II | NCT04194801 | Recruitment Status: Unknown;  First Posted: December 11, 2019；  Last Update Posted: April 9, 2020. | BLU-554 combinates with cs1001 (Sugemalimab, PD-L1 monoclonal antibody) is undergoing a multicenter clinical trial in China. |
| 3 | H3B-6527 | 1702259-66-2 | 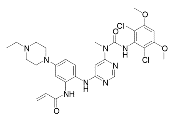 | H3Biomedicine | Advanced HCC | PO | Phase I | NCT02834780 | Recruitment Status: Completed;  First Posted: July 15, 2016;  Last Update Posted: April 6, 2022. | In preclinical trials, H3B-A6527 was shown to be effective in inhibiting tumor growth in HCC Xenograft transplantation model activated by abnormal FGF19-FGFR4 signals. Preclinical studies have shown that FGF19 expression is a predictive biomarker of H3B6527 response. In addition, in the HCC Xenograft transplantation model, H3B-6527 combined with Palbociclib could inhibit tumor growth synergistically (Joshi et al. 2017). |
| 4 | U3-1784 | —— | —— | Daiichi Sankyo Inc. | Advanced HCC | IV | Phase I | NCT02690350 | Recruitment Status: Terminated (Program discontinued for business reasons (not safety);  First Posted: February 24, 2016;  Last Update Posted: May 16, 2018. | The effect of u3-1784 monotherapy was similar to that of sorafenib on the growth inhibition of HCC, but there were no side effects such as weight loss caused by sorafenib. Therefore, the combination of U3-1784 and sorafenib is possible, which does not increase the toxic effect of sorafenib and further reduces the growth of tumors (Bartz et al. 2019). |
|  | INCB-62079 | —— | —— | Incyte Corporation | HCC | PO | Phase I | NCT03144661 | Recruitment Status: Terminated;  First Posted: May 9, 2017;  Last Update Posted: July 15, 2020. | INCB-62079 entered phase I trial in 2017 but was terminated for business strategic consideration (Zheng J. et al. 2022). |
| 6 | ICP-105 | —— | —— | InnoCare Pharma Tech Co. | HCC; Solid Tumor | PO | Phase I | NCT03642834 | Recruitment Status: Recruiting;  First Posted: August 22, 2018;  Last Update Posted: July 29, 2021. | ICP-105 is a highly specific FGFR4 inhibitor, with excellent target selectivity and good drug formation. It has a significant anti-tumor effect in HCC animal models with abnormal FGFR4 signaling pathway. The company also plans to explore the combination of ICP-105 and immune checkpoint inhibitor (ICI) to treat advanced HCC with excessive activation of FGFR4 pathway. (Data from company's announcement) |
| 7 | ZSP-1241 | —— | —— | Guangdong Zhongsheng Pharmaceutical Co. | HCC; Solid Tumor | PO | Phase I | NCT03734926 | Recruitment Status: Recruiting;  First Posted: November 8, 2018;  Last Update Posted: July 22, 2020. | Preclinical research results showed that ZSP-1241 showed significant antitumor effects on a variety of liver cancer, gastric cancer CDX or PDX models, and had synergistic effects with sorafenib. (Data from company's announcement) |
| 8 | HS-236 | —— | —— | Hisunpharm | HCC; Solid Tumor | PO | Phase I | CXHL2000217,CXHL2000218; | In December 2020, the phase I trial of advanced solid tumors was launched. | No information. |
| 9 | BPI-43487 | —— | —— | Bettapharma | HCC; middle and advanced solid tumors | PO | Phase I | CXHL2000011,CXHL2000010; | In March 2021, it was listed in the phase I clinical development stage of advanced solid tumors on the company's official website. | No information. |
| 10 | SY-4798 | —— | —— | Shouyao Holdings (Beijing) Co. | HCC; Advanced solid tumors | PO | Phase I | CXHL2000656 | In April 2021, China launched the phase I trial of advanced solid tumors. | SY-4798, as a new generation of FGFR4 small molecule inhibitor with high selectivity, high activity and irreversibility, has shown excellent therapeutic effects on FGFR4 positive tumors in preclinical studies. Preclinical studies have shown that the antitumor activity of SY-4798 in animals is 5-10 times higher than BLU-554; the efficacy and safety window are more than 10 times higher (Data from company's announcement). |
| 11 | BLU-9931 | 1538604-68-0 | 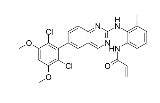 | Blueprint Medicines | HCC | —— | Preclinical stage | Preclinical stage | Preclinical stage | Oral administration of BLU-9931 can induce apoptosis of HCC cells in nude mice and significantly inhibit the growth of Hep3B tumor xenografts (Hagel et al. 2015). |
| 12 | AZ709 | —— | —— | AstraZeneca | HCC | —— | Preclinical stage | Preclinical stage | Preclinical stage | AZ709 has limited antiproliferative effect on a group of HCC cells in vitro. AZ709 inhibited the phosphorylation of FGFR4 and downstream substrates, such as apoptosis related p-MAPK (Lorraine M. et al. 2013). |

**TABLE S2. The information of drugs.**

| No. | Drug name | CAS No. | Company | Catalog No. | Target from commodity website | Molecular Weight | Solvent |
| --- | --- | --- | --- | --- | --- | --- | --- |
| 1 | FGF401 (Roblitinib) | 1708971-55-4 | CASYMCHEM | EVER4010007 | FGFR4 | 506.56 | DMSO |
| 2 | BLU-554 (Fisogatinib) | 1707289-21-1 | MedChemExpress | HY-100492 | FGFR4 | 503.38 | DMSO |
| 3 | Geldanamycin | 30562-34-6 | MedChemExpress | HY-15230 | HSP; Bacterial; Influenza Virus; Antibiotic | 560.64 | DMSO |
| 4 | Imatinib | 152459-95-5 | MedChemExpress | HY-15463 | Bcr-Abl; PDGFR; c-Kit; SARS-CoV; Autophagy | 493.6 | DMSO |
| 5 | LY-294002 | 154447-36-6 | MedChemExpress | HY-10108 | PI3K; Casein Kinase; DNA-PK; Apoptosis; Autophagy | 307.34 | DMSO |
| 6 | Parthenolide | 20554-84-1 | MedChemExpress | HY-N0141 | NF-κB; Autophagy; Mitophagy; Apoptosis | 248.32 | DMSO |
| 7 | Tanespimycin | 75747-14-7 | MedChemExpress | HY-10211 | HSP; Autophagy; Mitophagy; Bacterial; Apoptosis; Antibiotic | 585.69 | DMSO |
| 8 | Trichostatin A | 58880-19-6 | MedChemExpress | HY-15144 | Cell Cycle/DNA Damage; Epigenetics | 302.37 | DMSO |
| 9 | Vorinostat | 149647-78-9 | MedChemExpress | HY-10221 | HDAC; Autophagy; Mitophagy; Filovirus; Apoptosis; HPV | 264.32 | DMSO |

**TABLE S3. Antibody information for IHC, IF and WB assay.**

| Antibodies name | Company | Catalog No. | Application |
| --- | --- | --- | --- |
| FGFR4 | Abclonal | A9197 | IHC |
| FGFR4 | Cell Signaling Technology | 2894 | IF |
| FGF19 | Abcam | ab225942 | IHC |
| KLB | Abcam | ab106794 | IHC |
| p-ERK 1/2 | Abclonal | AP0974 | WB |
| ERK 1/2 | Abclonal | AP4782 | WB |
| p-AKT (T308) | Cell Signaling Technology | 5106S | WB |
| AKT | Cell Signaling Technology | 9272S | WB |
| GAPDH | Servicebio | GB11002 | WB |
| HRP labeled goat anti rabbit IgG (H+L) | Beyotime | A0208 | WB |
| HRP labeled goat anti mouse IgG (H+L) | Beyotime | A0216 | WB |

**TABLE S4. The primer sequences of PCR assay.**

| Primer name | Direction | Primer sequences (5’ - 3’) |
| --- | --- | --- |
| FGFR4 | Forward | GAGGGGCCGCCTAGAGATT |
|  | Reverse | GAGGGGCCGCCTAGAGATT |
| FGF19 | Forward | CGGAGGAAGACTG TGCTTTCG |
|  | Reverse | CTCGGATCGGTACACATTGTAG |
| KLB | Forward | TCTG TCATCCTGTCAGCACTT |
|  | Reverse | CCAGTCCCAATACCCCAGAAAAA |
| β-actin | Forward | ATCGTCCACCGCAAATGCTTCTA |
|  | Reverse | AGCCATGCCAATCTCATCTTGTT |

**TABLE S5. Patient information for IHC assay.**

| No. | Surgical date | Gender | Age | AFP (ng/ml) | HBsAg | Maximum tumor diameter （cm） | Tumor Number | Tumor differentiation degree | MVI | Lymph node metastasis | Liver cirrhosis | FGF19 IHC quantity | FGFR4 IHC quantity | KLB IHC quantity |
| --- | --- | --- | --- | --- | --- | --- | --- | --- | --- | --- | --- | --- | --- | --- |
| 01 | 2019/9/5 | F | 55 | 6.61 | + | 6.5 | 1 | High-Medium | M0 | - | + | L | M | L |
| 02 | 2020/1/23 | M | 65 | 79.54 | - | 6.3 | 1 | Medium-Low | M2 | - | + | N | L | M |
| 03 | 2020/5/22 | M | 61 | 4.05 | + | 3 | 1 | Medium | M0 | - | + | L | L | H |
| 04 | 2020/5/12 | M | 64 | 3.37 | + | 5.5 | 1 | Medium | M0 | - | + | M | N | M |
| 05 | 2019/12/30 | M | 50 | 94.24 | + | 15 | n>5 | Medium | M0 | - | + | N | N | N |
| 06 | 2019/11/4 | M | 51 | >1956.7 | + | 2 | n>5 | Medium | M2 | - | + | N | L | N |
| 07 | 2020/5/18 | M | 65 | 2.48 | + | 5.5 | 2 | Medium | M0 | - | + | N | M | N |
| 08 | 2020/1/12 | M | 47 | 3.02 | + | 7.5 | 3 | Medium-Low | M0 | - | + | H | M | M |
| 09 | 2020/4/20 | M | 66 | <2 | + | 2.6 | 1 | Medium | M0 | - | + | H | M | H |
| 10 | 2020/4/26 | M | 69 | >1956.7 | - | 15.5 | 1 | Medium | M2 | - | + | H | H | H |
| 11 | 2019/12/30 | M | 46 | 1703.29 | - | 2 | 1 | Medium | M0 | - | - | N | H | N |
| 12 | 2020/8/31 | M | 52 | <2 | + | 15 | 3 | Medium | M0 | - | + | N | N | N |
| 13 | 2020/6/28 | M | 50 | 6.45 | + | 2.5 | 1 | Medium | M0 | - | + | N | H | N |
| 14 | 2020/10/15 | F | 44 | 21.95 | + | 1.3 | 1 | Medium | M0 | - | + | L | H | N |
| 15 | 2021/4/16 | M | 77 | 11.04 | + | 12 | 1 | Medium | M0 | - | + | N | N | N |
| 16 | 2021/5/19 | M | 58 | <2 | + | 3 | 1 | Medium | M0 | - | + | L | H | N |
| 17 | 2021/5/27 | M | 65 | >1956.7 | + | 1 | 1 | Medium-Low | M2 | - | + | N | H | N |
| 18 | 2022/3/4 | M | 47 | 55.34 | + | 1.7 | 1 | Medium | M0 | - | + | M | H | L |
| 19 | 2021/7/5 | M | 50 | 34.5 | - | 2.5 | n>5 | Medium | M0 | - | + | N | H | N |
| 20 | 2022/1/29 | M | 73 | 3.45 | - | 8 | 1 | Medium | M0 | - | + | N | H | N |
| 21 | 2022/2/28 | M | 48 | 19.04 | + | 2 | n>5 | Medium | M0 | - | + | L | M | L |
| 22 | 2022/4/29 | M | 51 | 8.24 | - | 4.7 | n>5 | Medium | M0 | - | + | N | N | N |
| 23 | 2022/5/17 | M | 41 | 26.03 | + | 18 | 1 | Medium | M2 | + | + | M | M | N |
| 24 | 2021/11/16 | M | 67 | 36.58 | + | 1.2 | 3 | Medium | M0 | - | + | M | M | N |
| 25 | 2022/4/26 | M | 48 | 3.25 | - | 5.5 | n>5 | Medium | M2 | - | + | N | N | N |
| 26 | 2022/1/18 | F | 56 | 7.25 | - | 5 | 1 | Medium | M0 | - | + | N | H | L |
| 27 | 2022/8/1 | F | 59 | 9.43 | - | 4 | 1 | Medium | M0 | - | + | N | L | N |
| 28 | 2022/1/7 | F | 72 | 2.32 | - | 6.5 | 1 | Medium | M0 | - | + | M | N | L |
| 29 | 2022/6/14 | M | 48 | 3.27 | + | 3.5 | n>5 | Medium | M0 | - | + | M | M | L |
| 30 | 2022/7/11 | M | 51 | <2 | - | 2.7 | 2 | Medium | M0 | - | + | N | N | N |
| 31 | 2022/2/8 | M | 50 | 220.63 | + | 5 | 1 | Medium | M1 | - | + | N | N | N |
| 32 | 2022/4/27 | M | 14 | 9.85 | - | 11 | 1 | Medium | M2 | - | + | L | M | N |
| 33 | 2022/4/14 | M | 58 | 81.55 | + | 2.2 | 1 | Medium | M0 | + | + | M | L | N |
| 34 | 2022/5/10 | M | 58 | 6.34 | + | 4 | 3 | Medium | M0 | - | + | M | M | M |
| 35 | 2022/7/20 | M | 45 | >1956.7 | - | 4.5 | n>5 | Medium | M0 | - | + | N | L | L |

Gender: F: female; M: male.

IHC quantity: H: > 75%; M: 25%-75%; L: < 25%; N: None.

MVI (microvascular invasion): M0: Not found; M1: Low risk; M2: High risk.

**TABLE S6. Matching potential combination drugs according to co-expressed genes.**

| No. | cMAP name | Mean | N | Enrichment | p-value |
| --- | --- | --- | --- | --- | --- |
| 1 | Vorinostat | -0.608 | 12 | -0.66 | 0 |
| 2 | 15-delta prostaglandin J2 | -0.471 | 15 | -0.621 | 0 |
| 3 | Trichostatin A | -0.547 | 182 | -0.556 | 0 |
| 4 | Tanespimycin | -0.532 | 62 | -0.505 | 0 |
| 5 | LY-294002 | -0.317 | 61 | -0.288 | 0.00006 |
| 6 | Geldanamycin | -0.491 | 15 | -0.535 | 0.00018 |
| 7 | Alvespimycin | -0.479 | 12 | -0.566 | 0.0004 |
| 8 | Methyldopate | 0.546 | 4 | 0.839 | 0.00105 |
| 9 | Clotrimazole | -0.566 | 5 | -0.739 | 0.00244 |
| 10 | Mecamylamine | -0.66 | 3 | -0.885 | 0.00298 |
| 11 | 4,5-dianilinophthalimide | -0.8 | 2 | -0.963 | 0.00304 |
| 12 | Thiostrepton | -0.653 | 4 | -0.801 | 0.00304 |
| 13 | Imatinib | 0.631 | 2 | 0.951 | 0.00439 |
| 14 | Methapyrilene | -0.604 | 4 | -0.775 | 0.00523 |
| 15 | SC-19220 | 0.482 | 4 | 0.77 | 0.00543 |
| 16 | Prochlorperazine | -0.375 | 16 | -0.41 | 0.00606 |
| 17 | Blebbistatin | -0.728 | 2 | -0.94 | 0.00765 |
| 18 | Butamben | 0.345 | 4 | 0.746 | 0.00788 |
| 19 | Parthenolide | -0.598 | 4 | -0.745 | 0.00839 |
| 20 | NS-398 | -0.63 | 3 | -0.839 | 0.00841 |

**TABLE S7. Matching potential combination drugs according to pathways.**

| No. | cMap name | Mean | N | Enrichment | p-value |
| --- | --- | --- | --- | --- | --- |
| 1 | Felbinac | -0.823 | 4 | -0.884 | 0.0004 |
| 2 | Cicloheximide | -0.679 | 4 | -0.85 | 0.00095 |
| 3 | Mercaptopurine | 0.782 | 2 | 0.965 | 0.00207 |
| 4 | Aciclovir | -0.525 | 6 | -0.688 | 0.00222 |
| 5 | Eldeline | 0.381 | 6 | 0.688 | 0.00238 |
| 6 | Monensin | -0.416 | 6 | -0.679 | 0.003 |
| 7 | Pirinixic acid | 0.605 | 5 | 0.732 | 0.00312 |
| 8 | N-acetyl-L-leucine | 0.476 | 4 | 0.797 | 0.00328 |
| 9 | 3-nitropropionic acid | 0.31 | 4 | 0.793 | 0.00362 |
| 10 | Phenoxybenzamine | 0.587 | 4 | 0.79 | 0.0039 |
| 11 | Lovastatin | -0.578 | 4 | -0.79 | 0.00398 |
| 12 | Napelline | 0.344 | 4 | 0.787 | 0.004 |
| 13 | Luteolin | 0.391 | 4 | 0.784 | 0.00412 |
| 14 | Nadolol | -0.312 | 4 | -0.776 | 0.00519 |
| 15 | Furazolidone | -0.651 | 4 | -0.771 | 0.00561 |
| 16 | Harmol | 0.443 | 4 | 0.765 | 0.00579 |
| 17 | Josamycin | -0.505 | 5 | -0.695 | 0.00581 |
| 18 | Azlocillin | 0.461 | 4 | 0.764 | 0.00591 |
| 19 | Eticlopride | -0.502 | 4 | -0.763 | 0.00649 |
| 20 | Lycorine | -0.471 | 5 | -0.685 | 0.00717 |

**TABLE S8. The information of combination drugs with FGFR4 inhibitors.**

| Drug | Structure | Target | Pathway | The latest progress related to HCC |
| --- | --- | --- | --- | --- |
| Geldanamycin | 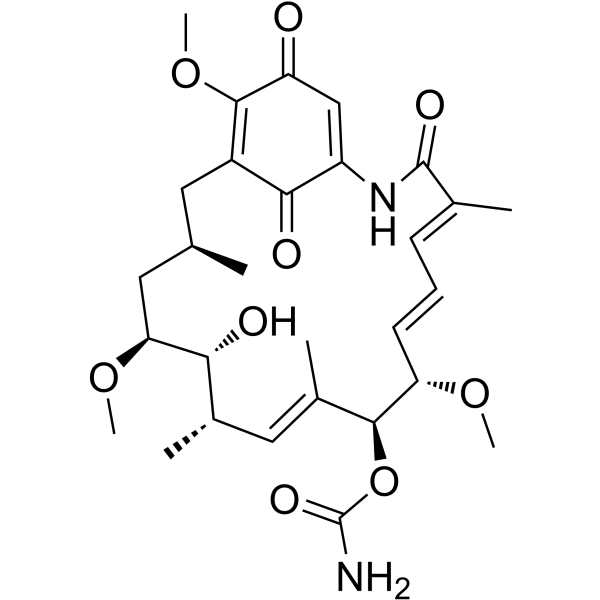 | Endoplasmin,Heat shock protein HSP 90-alpha  Heat shock protein HSP 90-beta | Protein processing in endoplasmic reticulum  PI3K-Akt signaling pathway  Antigen processing and presentation  NOD-like receptor signaling pathway  Progesterone-mediated oocyte maturation  Estrogen signaling pathway  Pathways in cancer | Geldanamycin, as the most representative Hsp90 inhibitor, has great potential to target a variety of cancer signaling pathways including HCC in the absence of drug interaction, targeting effect, systemic toxicity or multidrug resistance complexity (Bohonowych, Gopal, and Isaacs 2010). |
| Imatinib | 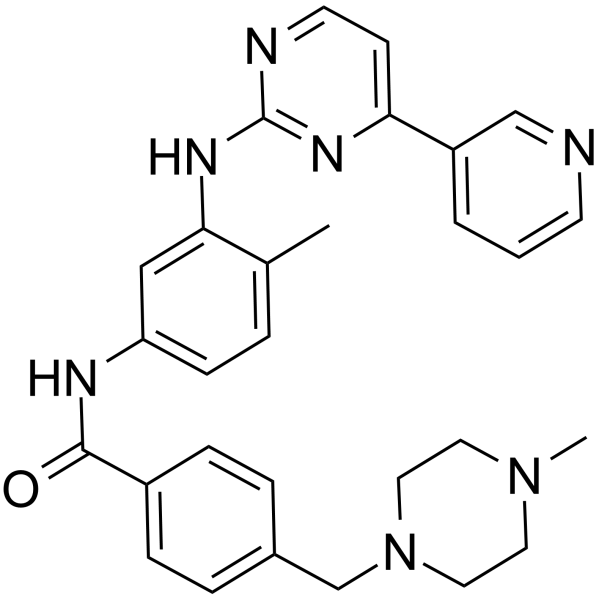 | Breakpoint cluster region protein  Mast/stem cell growth factor receptor Kit  RET proto-oncogene  High affinity nerve growth factor receptor  Macrophage colony-stimulating factor 1 receptor  Platelet-derived growth factor receptor alpha  Epithelial discoidin domain-containing receptor 1  Tyrosine-protein kinase ABL1  Platelet-derived growth factor receptor beta  Discoidin domain-containing receptor 2 | Ras signaling pathway  Rap1 signaling pathway  Cytokine-cytokine receptor interaction  Endocytosis  PI3K-Akt signaling pathway  Hematopoietic cell lineage  Melanogenesis  Pathways in cancer  Acute myeloid leukemia  Central carbon metabolism in cancer | Imatinib significantly inhibited tumor growth of HCC patient derived xenografts (PDXs) (Nazzal et al. 2020). Imatinib has antitumor effect on HCC by inhibiting Akt phosphorylation and attenuating autophagy in HCC cells; The combination of imatinib and sorafenib showed synergistic inhibition on HCC (Xiao et al. 2021). |
| LY-294002 | 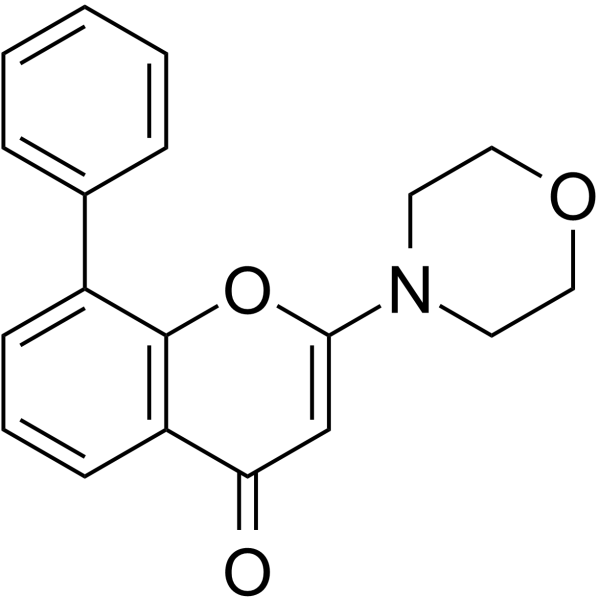 | Phosphatidylinositol 4,5-bisphosphate 3-kinase catalytic subunit gamma isoform  Serine/threonine-protein kinase pim-1 | Fc epsilon RI signaling pathway  Fc gamma R-mediated phagocytosis  TNF signaling pathway  Leukocyte transendothelial migration  Neurotrophin signaling pathway  Cholinergic synapse  Inflammatory mediator regulation of TRP channels  Regulation of actin cytoskeleton  Insulin signaling pathway  Progesterone-mediated oocyte maturation  Estrogen signaling pathway  Prolactin signaling pathway  Thyroid hormone signaling pathway  Oxytocin signaling pathway  Regulation of lipolysis in adipocytes  Type II diabetes mellitus  Chemokine signaling pathway  cAMP signaling pathway  cGMP-PKG signaling pathway  Rap1 signaling pathway  Ras signaling pathway  ErbB signaling pathway  Inositol phosphate metabolism  Pathways in cancer  Viral carcinogenesis  Proteoglycans in cancer  MicroRNAs in cancer  B cell receptor signaling pathway  Hepatitis C  Toll-like receptor signaling pathway  Carbohydrate digestion and absorption  Aldosterone-regulated sodium reabsorption  Non-alcoholic fatty liver disease (NAFLD)  Central carbon metabolism in cancer  Choline metabolism in cancer  Hepatitis B  FoxO signaling pathway  Phosphatidylinositol signaling system  Sphingolipid signaling pathway  mTOR signaling pathway  PI3K-Akt signaling pathway  AMPK signaling pathway  Apoptosis  Adrenergic signaling in cardiomyocytes  VEGF signaling pathway  Osteoclast differentiation  Focal adhesion  Signaling pathways regulating pluripotency of stem cells  Platelet activation  HIF-1 signaling pathway  Jak-STAT signaling pathway  Natural killer cell mediated cytotoxicity  T cell receptor signaling pathway | LY-294002 treatment of HCC cells can significantly reduce tumor cell viability and promote apoptosis; At the same time, LY-294002 can also reduce the ability of HCC cell migration and invasion (Ma et al. 2014). |
| Parthenolide | 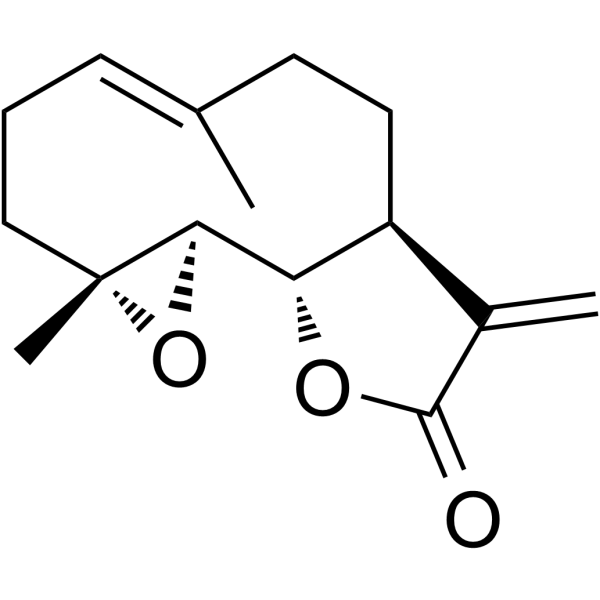 | Inhibitor of nuclear factor kappa-B kinase beta (IKKB)  Nuclear factor NF-kappa-B (NFKB) | Neurotrophin signaling pathway  Non-alcoholic fatty liver disease (NAFLD)  Adipocytokine signaling pathway  RIG-I-like receptor signaling pathway  Cytosolic DNA-sensing pathway  T cell receptor signaling pathway  Insulin signaling pathway  NF-kappa B signaling pathway  B cell receptor signaling pathway  TNF signaling pathway  MAPK signaling pathway  Ras signaling pathway  Chemokine signaling pathway  Toll-like receptor signaling pathway  FoxO signaling pathway  mTOR signaling pathway  PI3K-Akt signaling pathway  Apoptosis  Osteoclast differentiation  NOD-like receptor signaling pathway  Hepatitis C  Hepatitis B  Pathways in cancer  Viral carcinogenesis  MicroRNAs in cancer | Parthenolide can promote apoptosis of three HCC cell lines (HepG2, Hep3B, SK-Hep-1) (Carlisi et al. 2011). In addition, Research in vitro and in vivo have shown that Parthenolide and Sorafenib have synergistic effects, which can inhibit the proliferation and migration of HCC cells (Liang et al. 2020). |
| Tanespimycin | 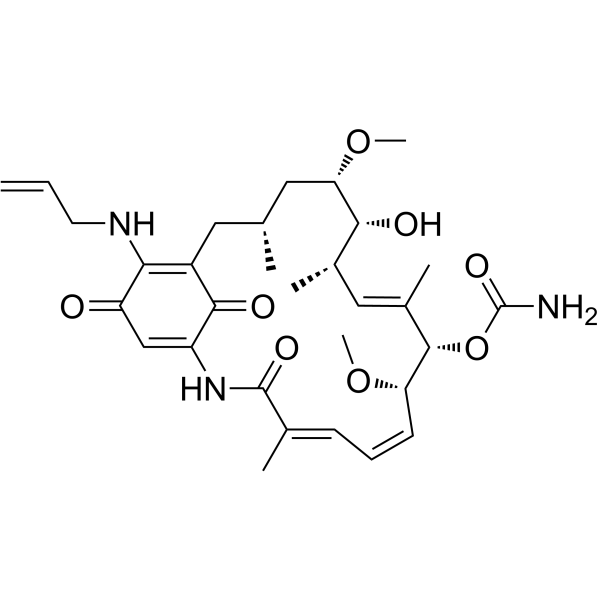 | Heat shock protein HSP 90-alpha  Heat shock protein HSP 90-beta | Protein processing in endoplasmic reticulum  Pathways in cancer  Estrogen signaling pathway  Progesterone-mediated oocyte maturation  NOD-like receptor signaling pathway  Antigen processing and presentation  PI3K-Akt signaling pathway | Tanespimycin mediated Hsp90 inhibition can induce G2 / M cell cycle arrest and apoptosis, thus inhibiting the growth of HCC cell lines and tumors in vivo (Watanabe et al. 2009). |
| Trichostatin A | 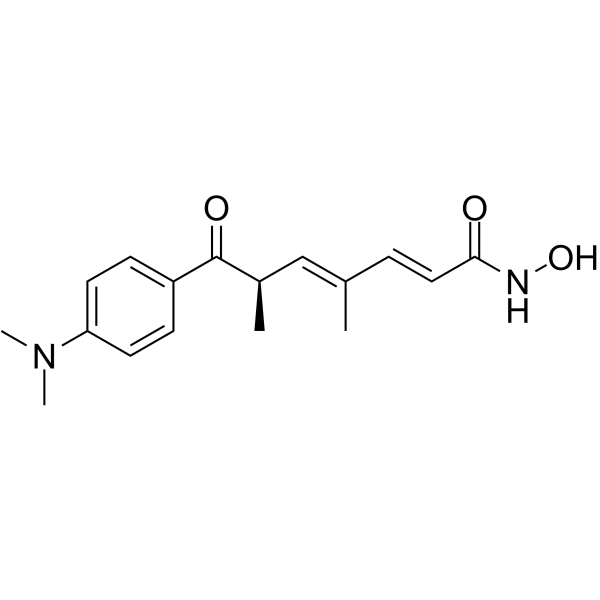 | Histone deacetylase 7  Histone deacetylase 8  Acetoin utilization protein Caspase-8  Prostaglandin E2 Receptors (Protein Group)  Tumor necrosis factor  Histone deacetylase (HDAC) | —— | In HCC cells (HCCLM3, MHCC97H and MHCC97l ), the HDAC inhibitor Trichostatin A can induce apoptosis and inhibit cell growth through mitochondrial / endogenous and cytoplasmic / exogenous apoptosis pathways (Sanaei and Kavoosi 2021). |
| Vorinostat | 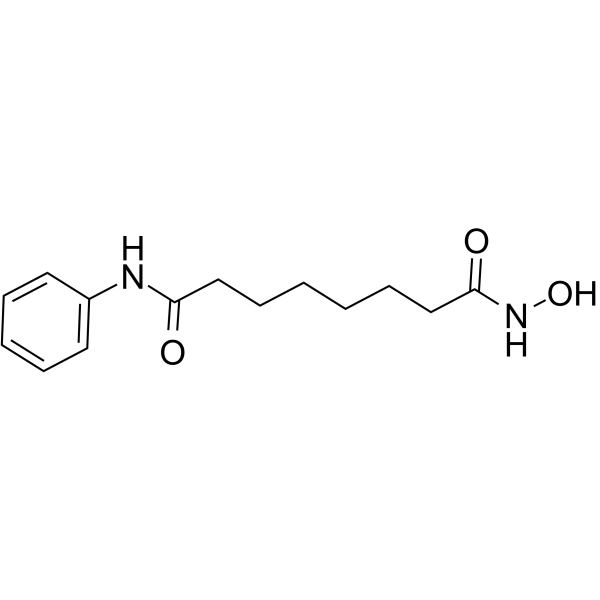 | Histone deacetylase 1  Histone deacetylase 2  Histone deacetylase 3  Histone deacetylase 6  Histone deacetylase 8  Acetoin utilization protein  Histone deacetylase (Protein Group)  Histone deacetylase 1 (HDAC1) | Notch signaling pathway  MicroRNAs in cancer  Viral carcinogenesis  Thyroid hormone signaling pathway  Amphetamine addiction  Alcoholism  Transcriptional misregulation in cancer  Cell cycle  Pathways in cancer | The combination of Vorinostat and Oxaliplatin has a synergistic effect in HCC cells, which can inhibit cell growth, induce G2 / M phase arrest, and lead to apoptosis. And studies have shown that Vorinostat can enhance the anticancer effect of oxaliplatin on HCC cells (Liao et al. 2018). In addition, Vorinostat can also enhance the anti-HCC activity of other chemotherapeutic drugs, including Sorafenib, 5-Fluorouracil and so on (Li et al. 2018). |

**TABLE S9. IC_30_ and IC_50_ of drugs.**

|  | Huh-7 | | Hep3B | |
| --- | --- | --- | --- | --- |
| Concentration (μM) | IC_30_ | IC_50_ | IC_30_ | IC_50_ |
| FGF401 | 48.63±7.20 | 135.89±13.11 | 80±3.77 | 100±4.71 |
| BLU-554 | 22.18±0.52 | 102.02±12.45 | 20.9±0.49 | 36.79±2.63 |
| Geldanamycin | 11.54±0.54 | 21.15±2.04 | 1.7±0.21 | 3.15±0.23 |
| Imatinib | 13.59±0.64 | 19.84±1.91 | 3.34±0.00 | 5.66±0.40 |
| LY-294002 | 14.66±1.05 | 77.18±3.64 | 6.2±0.29 | 47.1±3.37 |
| Parthenolide | 41.96±1.98 | 52.66±2.48 | 5.66±0.40 | 6.8±0.32 |
| Tanespimycin | 32.68±4.84 | 49.6±2.34 | 1.7±0.04 | 25.81±1.85 |
| Trichostatin A | 50.8±2.39 | 130.08±9.30 | 5.53±0.53 | 22.63±0.53 |
| Vorinostat | 99.61±7.12 | 179.6±17.33 | 6.2±0.14 | 56.12±4.01 |

**TABLE S10. Cell viability of the combined schemes.**

|  |  | Huh-7 | | | | Hep3B | | | |
| --- | --- | --- | --- | --- | --- | --- | --- | --- | --- |
|  |  | FGF401 | | BLU-554 | | FGF401 | | BLU-554 | |
|  |  | IC_30_ | IC_50_ | IC_30_ | IC_50_ | IC_30_ | IC_50_ | IC_30_ | IC_50_ |
| Geldanamycin | IC_30_ | 0.44±0.02 | 0.17±0.01 | 0.48±0.00 | 0.12±0.01 | 0.34±0.02 | 0.18±0.03 | 0.4±0.00 | 0.29±0.00 |
|  | IC_50_ | 0.42±0.04 | 0.17±0.02 | 0.48±0.01 | 0.13±0.01 | 0.29±0.01 | 0.15±0.00 | 0.4±0.00 | 0.28±0.00 |
| Imatinib | IC_30_ | 0.95±0.04 | 0.19±0.03 | 0.59±0.04 | 0.07±0.01 | 0.69±0.03 | 0.45±0.01 | 0.87±0.04 | 0.39±0.01 |
|  | IC_50_ | 0.32±0.04 | 0.04±0.00 | 0.39±0.04 | 0.05±0.01 | 0.51±0.02 | 0.35±0.02 | 0.45±0.01 | 0.19±0.01 |
| LY-294002 | IC_30_ | 0.54±0.04 | 0.39±0.03 | 0.56±0.05 | 0.2±0.00 | 0.42±0.03 | 0.28±0.02 | 0.56±0.00 | 0.18±0.00 |
|  | IC_50_ | 0.47±0.03 | 0.31±0.05 | 0.43±0.02 | 0.06±0.02 | 0.23±0.03 | 0.17±0.02 | 0.29±0.01 | 0.02±0.01 |
| Parthenolide | IC_30_ | 0.4±0.04 | 0.14±0.03 | 0.62±0.06 | 0.15±0.03 | 0.4±0.03 | 0.23±0.02 | 0.72±0.01 | 0.47±0.00 |
|  | IC_50_ | 0.16±0.02 | 0.03±0.00 | 0.32±0.01 | 0.03±0.01 | 0.13±0.01 | 0.08±0.02 | 0.29±0.00 | 0.01±0.00 |
| Tanespimycin | IC_30_ | 0.52±0.03 | 0.25±0.03 | 0.53±0.01 | 0.36±0.01 | 0.56±0.04 | 0.38±0.06 | 0.63±0.02 | 0.35±0.00 |
|  | IC_50_ | 0.42±0.02 | 0.16±0.02 | 0.4±0.03 | 0.37±0.02 | 0.36±0.04 | 0.2±0.02 | 0.5±0.02 | 0.18±0.01 |
| Trichostatin A | IC_30_ | 1.13±0.03 | 0.32±0.01 | 0.58±0.05 | 0.29±0.04 | 0.51±0.03 | 0.41±0.02 | 0.7±0.01 | 0.38±0.01 |
|  | IC_50_ | 0.87±0.05 | 0.34±0.02 | 0.43±0.02 | 0.3±0.03 | 0.34±0.02 | 0.27±0.01 | 0.47±0.00 | 0.21±0.01 |
| Vorinostat | IC_30_ | 0.68±0.01 | 0.39±0.00 | 0.48±0.01 | 0.25±0.01 | 0.53±0.07 | 0.4±0.02 | 0.7±0.02 | 0.49±0.02 |
|  | IC_50_ | 0.58±0.02 | 0.4±0.01 | 0.42±0.05 | 0.21±0.01 | 0.37±0.02 | 0.33±0.03 | 0.56±0.04 | 0.2±0.00 |

**TABLE S11. Combination index (CI) of the combined schemes.**

|  |  | Huh-7 | | | | Hep3B | | | |
| --- | --- | --- | --- | --- | --- | --- | --- | --- | --- |
|  |  | FGF401 | | BLU-554 | | FGF401 | | BLU-554 | |
|  |  | IC_30_ | IC_50_ | IC_30_ | IC_50_ | IC_30_ | IC_50_ | IC_30_ | IC_50_ |
| Geldanamycin | IC_30_ | 0.79±0.02 | 0.75±0.01 | 0.73±0 | 0.62±0.01 | 1.15±0.02 | 1.07±0.03 | 0.96±0.00 | 1.04±0.00 |
|  | IC_50_ | 1.18±0.05 | 1.08±0.02 | 1.17±0.01 | 0.95±0.01 | 1.54±0.01 | 1.39±0.00 | 1.41±0.00 | 1.43±0.00 |
| Imatinib | IC_30_ | — | 0.82±0.02 | 1.1±0.04 | 0.5±0.01 | 1.96±0.03 | 1.45±0.01 | 3.7±0.15 | 1.15±0.01 |
|  | IC_50_ | 1±0.04 | 0.6±0.00 | 1±0.04 | 0.56±0.01 | 1.84±0.02 | 1.43±0.03 | 1.36±0.01 | 0.64±0.01 |
| LY-294002 | IC_30_ | 0.65±0.03 | 0.8±0.02 | 0.62±0.03 | 0.4±0.00 | 0.81±0.03 | 0.8±0.02 | 0.87±0.00 | 0.36±0.00 |
|  | IC_50_ | 1.19±0.04 | 1.03±0.05 | 0.9±0.02 | 0.45±0.01 | 0.83±0.03 | 0.81±0.02 | 0.67±0.01 | 0.24±0.00 |
| Parthenolide | IC_30_ | 0.97±0.04 | 0.83±0.02 | 1.39±0.08 | 0.79±0.02 | 1.47±0.03 | 1.29±0.03 | 2.11±0.02 | 1.73±0.00 |
|  | IC_50_ | 0.79±0.02 | 0.75±0.00 | 0.93±0.01 | 0.67±0.01 | 0.9±0.01 | 0.74±0.01 | 1.11±0.00 | 0.3±0.00 |
| Tanespimycin | IC_30_ | 1.04±0.03 | 0.95±0.03 | 0.93±0.01 | 1.13±0.01 | 0.95±0.04 | 0.9±0.05 | 1±0.02 | 0.7±0.00 |
|  | IC_50_ | 1.18±0.02 | 1.33±0.03 | 1.03±0.03 | 1.48±0.03 | 1.24±0.04 | 0.96±0.02 | 1.57±0.03 | 0.63±0.01 |
| Trichostatin A | IC_30_ | — | 0.77±0.01 | 0.88±0.04 | 0.66±0.03 | 1.08±0.03 | 0.98±0.02 | — | 0.8±0.01 |
|  | IC_50_ | — | 1.17±0.02 | 0.94±0.02 | 1±0.03 | 0.87±0.02 | 0.85±0.01 | 1.2±0.00 | 0.48±0.00 |
| Vorinostat | IC_30_ | 1.77±0.02 | 1.11±0.00 | 0.74±0.01 | 0.7±0.01 | 0.96±0.07 | 0.95±0.02 | 2.04±0.04 | 1.08±0.02 |
|  | IC_50_ | 1.58±0.03 | 1.49±0.01 | 1±0.05 | 0.84±0.01 | 1.13±0.02 | 1.17±0.03 | 2.32±0.09 | 0.58±0.00 |

**References**

Bartz, René, Keisuke Fukuchi, Toshiaki Ohtsuka, Tanja Lange, Katrin Gruner, Ichiro Watanabe, Shinko Hayashi, et al. 2019. ‘Preclinical Development of U3-1784, a Novel FGFR4 Antibody Against Cancer, and Avoidance of Its On-Target Toxicity’. *Molecular Cancer Therapeutics* 18 (10): 1832–43. https://doi.org/10.1158/1535-7163.MCT-18-0048.

Bohonowych, J. E., U. Gopal, and J. S. Isaacs. 2010. ‘Hsp90 as a Gatekeeper of Tumor Angiogenesis: Clinical Promise and Potential Pitfalls’. *Journal of Oncology* 2010: 412985. https://doi.org/20160818100439.

Carlisi, Daniela, Antonella D’Anneo, Liliana Angileri, Marianna Lauricella, Sonia Emanuele, Andrea Santulli, Renza Vento, and Giovanni Tesoriere. 2011. ‘Parthenolide Sensitizes Hepatocellular Carcinoma Cells to TRAIL by Inducing the Expression of Death Receptors through Inhibition of STAT3 Activation’. *Journal of Cellular Physiology* 226 (6): 1632–41. https://doi.org/10.1002/jcp.22494.

Hagel, Margit, Chandra Miduturu, Michael Sheets, Nooreen Rubin, Weifan Weng, Nicolas Stransky, Neil Bifulco, et al. 2015. ‘First Selective Small Molecule Inhibitor of FGFR4 for the Treatment of Hepatocellular Carcinomas with an Activated FGFR4 Signaling Pathway’. *Cancer Discovery* 5 (4): 424–37. https://doi.org/10.1158/2159-8290.CD-14-1029.

Joshi, Jaya Julie, Heather Coffey, Erik Corcoran, Jennifer Tsai, Chia-Ling Huang, Kana Ichikawa, Sudeep Prajapati, et al. 2017. ‘H3B-6527 Is a Potent and Selective Inhibitor of FGFR4 in FGF19-Driven Hepatocellular Carcinoma’. *Cancer Research* 77 (24): 6999–7013. https://doi.org/10.1158/0008-5472.CAN-17-1865.

Kim, R., D. Sarker, T. Macarulla, T. Yau, S.P. Choo, T. Meyer, A. Hollebecque, et al. 2017. ‘Phase 1 Safety and Clinical Activity of BLU-554 in Advanced Hepatocellular Carcinoma (HCC)’. *Annals of Oncology* 28 (September): v122. https://doi.org/10.1093/annonc/mdx367.

Li, Yang-Ling, Ning-Yu Zhang, Xiu Hu, Jia-Ling Chen, Ming-Jun Rao, Lin-Wen Wu, Qing-Yu Li, Bo Zhang, Wei Yan, and Chong Zhang. 2018. ‘Evodiamine Induces Apoptosis and Promotes Hepatocellular Carcinoma Cell Death Induced by Vorinostat via Downregulating HIF-1α under Hypoxia’. *Biochemical and Biophysical Research Communications* 498 (3): 481–86. https://doi.org/10.1016/j.bbrc.2018.03.004.

Liang, Pan, Hangyi Wu, Zhenhai Zhang, Shulong Jiang, and Huixia Lv. 2020. ‘Preparation and Characterization of Parthenolide Nanocrystals for Enhancing Therapeutic Effects of Sorafenib against Advanced Hepatocellular Carcinoma’. *International Journal of Pharmaceutics* 583 (June): 119375. https://doi.org/10.1016/j.ijpharm.2020.119375.

Liao, Bo, Yingying Zhang, Quan Sun, and Ping Jiang. 2018. ‘Vorinostat Enhances the Anticancer Effect of Oxaliplatin on Hepatocellular Carcinoma Cells’. *Cancer Medicine* 7 (1): 196–207. https://doi.org/10.1002/cam4.1278.

Lorraine M., Kerry S., Sarah C. et al. 2013 ‘Characterisation of AZ709, a Potent and Selective Inhibitor of Fibroblast Growth Factor Receptor 4 (FGFR4)’. *NCRI Cancer Conference Abstracts*. https://abstracts.ncri.org.uk/abstract/characterisation-of-az709-a-potent-and-selective-inhibitor-of-fibroblast-growth-factor-receptor-4-fgfr4-2/.

Ma, Jian, Shu-Li Xie, Ya-Jun Geng, Shuo Jin, Guang-Yi Wang, and Guo-Yue Lv. 2014. ‘In Vitro Regulation of Hepatocellular Carcinoma Cell Viability, Apoptosis, Invasion, and AEG-1 Expression by LY294002’. *Clinics and Research in Hepatology and Gastroenterology* 38 (1): 73–80. https://doi.org/10.1016/j.clinre.2013.06.012.

Nazzal, Mustafa, Subhayan Sur, Robert Steele, Mousumi Khatun, Tapas Patra, Nancy Phillips, John Long, Ranjit Ray, and Ratna B. Ray. 2020. ‘Establishment of a Patient-Derived Xenograft Tumor From Hepatitis C-Associated Liver Cancer and Evaluation of Imatinib Treatment Efficacy’. *Hepatology (Baltimore, Md.)* 72 (2): 379–88. https://doi.org/10.1002/hep.31298.

Sanaei, Masumeh, and Fraidoon Kavoosi. 2021. ‘Effects of Trichostatin A on the Intrinsic and Extrinsic Apoptotic Pathway, Cell Viability, and Apoptosis Induction in Hepatocellular Carcinoma Cell Lines’. *Gastroenterology and Hepatology from Bed to Bench* 14 (4): 323–33.

Watanabe, Go, Kevin E. Behrns, Jae-Sung Kim, and Robin D. Kim. 2009. ‘Heat Shock Protein 90 Inhibition Abrogates Hepatocellular Cancer Growth through Cdc2-Mediated G2/M Cell Cycle Arrest and Apoptosis’. *Cancer Chemotherapy and Pharmacology* 64 (3): 433–43. https://doi.org/10.1007/s00280-008-0888-2.

Xiao, Meng-Chao, Hui Qian, Chen-Kai Huang, Bai-Nan Zheng, Fang-Zhi Yan, Fang Liu, Xin Zhang, Shi-Jie Chen, Cheng Luo, and Wei-Fen Xie. 2021. ‘Imatinib Inhibits the Malignancy of Hepatocellular Carcinoma by Suppressing Autophagy’. *European Journal of Pharmacology* 906 (September): 174217. https://doi.org/10.1016/j.ejphar.2021.174217.

Zheng J, Zhang W, Li L, et al. Signaling Pathway and Small-Molecule Drug Discovery of FGFR: A Comprehensive Review. *Front Chem*. 2022;10:860985. Published 2022 Apr 14. doi:10.3389/fchem.2022.860985
